# Heterogeneous integration of ultrawide bandgap semiconductors for radio frequency power devices

**DOI:** 10.1126/sciadv.adw6167

**Published:** 2025-11-19

**Authors:** Hong Zhou, Min Zhou, Mingjie Xiang, Hehe Gong, Guangjie Gao, Chenlu Wang, Yachao Zhang, Kui Dang, Zhihong Liu, Jinfeng Zhang, HangMing Zhang, Yifan Wang, Han Wang, Mengwei Si, Yuhao Zhang, Yue Hao, Jincheng Zhang

**Affiliations:** ^1^ State Key Laboratory of Wide-Bandgap Semiconductor Devices and Integrated Technology, School of Microelectronics and Guangzhou Institute of Technology, Xidian University, Xi’an 710071, China.; ^2^ Center for Power Electronics Systems, Virginia Tech, Blacksburg, VA 24060, USA.; ^3^ Department of Electronic and Electrical Engineering and Centre for Advanced Semiconductors and Integrated Circuits, University of Hong Kong, Hong Kong, China.; ^4^ Department of Electronic Engineering, Shanghai Jiao Tong University, Shanghai 200240, China.

## Abstract

Ultrawide bandgap (UWBG) semiconductors offer high critical electric fields and saturation velocities ideal for radio frequency (rf) devices, but achieving both shallow-level doping and high thermal conductivity (*k*_T_) in a single material remains difficult. We demonstrate a scalable, exfoliation-based layer-transfer process to heterogeneously integrate gallium oxide (Ga_2_O_3_) thin films with shallow dopants onto high-*k*_T_ aluminum nitride (AlN) substrates. This method obviates ion implantation and interfacial dielectric layers used in conventional approaches. A large conduction band offset (3.4 electron volts) at the Ga_2_O_3_/AlN interface improves electron confinement in the Ga_2_O_3_ channel. T-gate rf power transistors achieve a maximum oscillation frequency of 90 gigahertz and output power densities of 4.6 watts per millimeter at 2 gigahertz and 4.1 watts per millimeter at 6 gigahertz—among the highest for UWBG devices. A minimal noise figure of 0.48 decibels at 8 gigahertz—among the lowest reported in this frequency range—further highlights the platform’s promise for next-generation rf applications.

## Teaser

A method bonds Ga_2_O_3_ to AlN, enabling radio frequency devices with enhanced power density and noise performance.

## INTRODUCTION

Radio frequency (rf) semiconductor devices are used ubiquitously in applications like telecommunication, consumer electronics, aerospace, defense, and health care, with a global market over $23 billion (https://fortunebusinessinsights.com/rf-semiconductor-market-110167). The overarching design target of many rf devices is to concurrently achieve high cutoff frequency (*f*_T_) and maximum oscillation frequency (*f*_max_), high output power density (*P*_out_), high efficiency, low noise, and high linearity. The frequency and power performance of rf devices can be substantially boosted by deploying wide bandgap (WBG) and ultrawide bandgap (UWBG) semiconductors, due to their superior properties including large bandgap, high critical electric field (*E*_C_), and high saturation velocity (ν_sat_) (*1*, *2*). The past two decades have witnessed the commercialization of WBG gallium nitride (GaN) rf power devices with a market size over $740 million (*3*). On the horizon, UWBG semiconductors such as gallium oxide (Ga_2_O_3_), aluminum nitride (AlN), boron nitride, and diamond promise a superior performance limit for rf devices (*4*). For example, the Johnson figure-of-merit (JFOM = *E*_C_^2^ × ν_sat_^2^/4π^2^), a widely used material indicator for rf device performance, is three times higher in Ga_2_O_3_ compared to GaN (*5*, *6*).

In addition to *E*_C_ and ν_sat_, the availability of shallow-level dopant and high thermal conductivity (*k*_T_) are also crucial to rf devices. The deep-level dopant can limit the device’s breakdown voltage (*BV*) and current density (*7*), and low *k*_T_ results in high junction temperature, both limiting the device power capacity. In addition, the relatively slow ionization of deep-level dopant could also limit the device switching frequency (*8*). Unfortunately, shallow dopants and low *k*_T_ are very challenging or even impossible to achieve in a single UWBG material (*2*). For example, Ga_2_O_3_ is known to offer shallow donors, large-area wafer, and a decent electron mobility, but it suffers a very low *k*_T_ that is only 1/6 of Si, 1/10 of GaN, and 1/20 of SiC (*5*). On the other hand, AlN and diamond have a high *k*_T_ but lack shallow-level dopants (*2*). Such material properties limit the rf device performance. For example, the state-of-the-art Ga_2_O_3_ rf transistors, despite having achieved a *f*_T_ of 10 to 27 GHz and *f*_max_ of 24 to 55 GHz (*9*–*11*), exhibit a limited *P*_out_ below 0.715 W/mm (*12*–*14*), which is much lower than the GaN counterpart.

A promising solution to the above challenge is combining the complementary properties of distinct UWBG materials through heterogeneous integration. To address the thermal limitation of Ga_2_O_3_, integration of Ga_2_O_3_ thin films on a variety of substrates including Si (*15*, *16*), SiC (*15*–*19*), GaN (*20*), and diamond (*21*–*24*) have been recently demonstrated. These integrations are primarily based on three approaches: (i) mechanical exfoliation and layer transfer (*20*–*22*, *24*, *25*), (ii) hydrogen (H) implantation–based ion cutting (*15*, *16*, *18*, *26*), and (iii) wafer fusion bonding (*17*, *23*, *27*, *28*). The Ga_2_O_3_-on-SiC rf transistors fabricated by ion implantation and bonding process have achieved a high *P*_out_ of 2.3 W/mm at *f* = 4 GHz (*19*), about three times higher than the best report on Ga_2_O_3_-on-Ga_2_O_3_.

However, the performance of Ga_2_O_3_-on-SiC rf transistors is still far from what the Ga_2_O_3_ material can provide, mainly due to several challenges facing the current heterogeneous integration techniques. First, the flakes produced in mechanical exfoliation usually have a size limited to submicrometers and highly uncontrollable, preventing the fabrication of large-area devices and microwave integrated circuits. Second, the ion cutting technique usually requires H implantation across the active device layer and high-temperature annealing, which can degrade the channel material (*29*). Third, many bonding techniques require an interfacial amorphous layer, which usually has a poor *k*_T_. Such a layer has been reported to generate an effective thermal boundary resistance up to 60 m^2^K/GW (*16*, *17*), accounting for up to 20% of the device’s total thermal resistance (*R*_T_). Last, the *E*_C_ of many host substrates (e.g., SiC) or interfacial layer is much lower than the channel material, leading to premature breakdown.

In this work, we develop a distinct heterogeneous integration technique that combines mechanical exfoliation, arrayed transfer, and wafer-level direct bonding. The Ga_2_O_3_ films are cleaved from the edge of a Ga_2_O_3_ substrate, ensuring a good uniformity of film size. These films are then transferred onto a substrate and placed in an array, followed by a wafer-level room-temperature bonding that reduces the thickness nonuniformity of the arrayed films. This method obviates the ion implantation, interfacial oxide, and high-temperature annealing required in many conventional heterogeneous integration approaches. The relatively good uniformity in geometries and thickness effectively upscales the total size of the exfoliated film arrays to the wafer scale, enabling the fabrication of large-area electronics such as interconnected devices and integrated circuits.

In addition, different from the Si and SiC substrates used in prior Ga_2_O_3_ integration studies, we select the AlN substrate, which offers a high *k*_T_ of ~3 W/(cm·K) and a high *E*_c_ up to 10 MV/cm (*30*) as well as a favorable band alignment to Ga_2_O_3_. Because of the ultralow electron affinity (0.6 eV) of AlN (*30*), a 3.4-eV high barrier forms at the Ga_2_O_3_/AlN interface, which is 2.5 eV higher than the Ga_2_O_3_-SiC interface. This barrier can enhance electron confinement in the thin Ga_2_O_3_ channel and favors the gate scaling in rf transistors. Leveraging these process and material innovations, the fabricated Ga_2_O_3_-on-AlN rf transistor sets performance records for *f*_max_, *P*_out_, and power-added efficiency (PAE) in UWBG rf devices. The average electric field and noise level is among the best in rf devices reported in all material systems, verifying the high material quality in this hetero-UWBG platform.

## RESULTS AND DISCUSSION

### Heterogeneous integration and material characterization

**Fig. 1.**
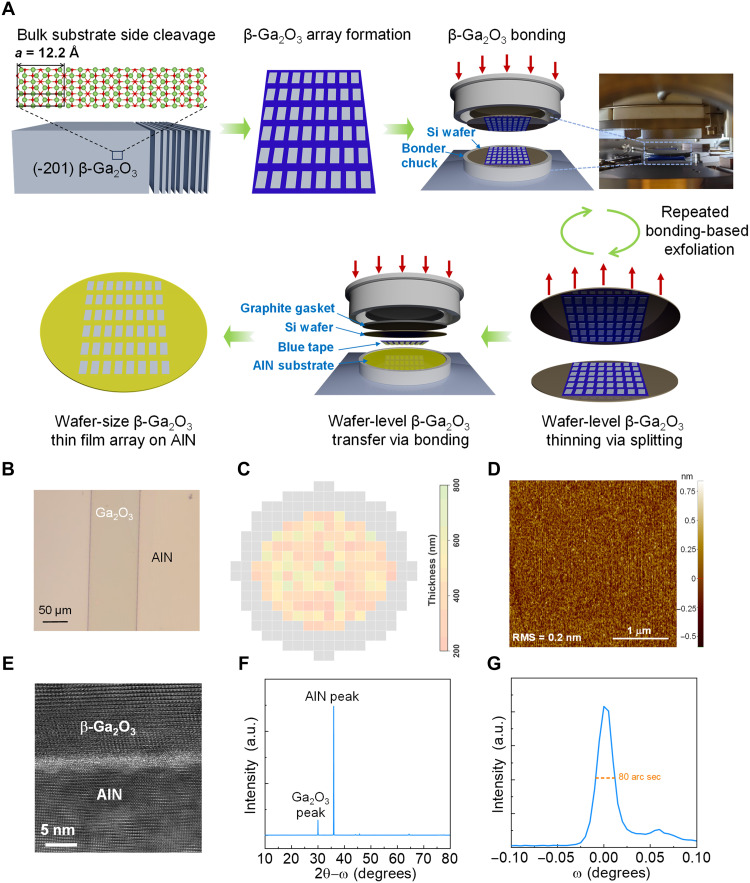
Heterogeneous integration process and material characterization. (**A**) Illustration of the Ga_2_O_3_/AlN heterogeneous integration process. The β-Ga_2_O_3_ belt or film with uniform geometry is cleaved from the substrate edge and then manually transferred onto the blue tape to form an array. The thickness of β-Ga_2_O_3_ films on blue tape is reduced by multiple wafer-level bonding process until the thickness reaches around 500 nm. The graphite/Si/β-Ga_2_O_3_/AlN stack is formed and loaded into the bonding chamber under a 3-hour bonding process to transfer the wafer-size β-Ga_2_O_3_ film array onto the AlN substrate. (**B**) Top-view optical microscopic image of part of a single β-Ga_2_O_3_ film on the AlN substrate. (**C**) Thickness mapping of 120 β-Ga_2_O_3_ films in a wafer-size array after being transferred onto the 1-inch AlN substrate. (**D**) Atomic force microscopy (AFM) image of the surface of β-Ga_2_O_3_ film transferred on the AlN substrate, revealing an atomic flat surface with an RMS roughness of 0.2 nm. (**E**) Zoomed-in and HRTEM image at the atomic interface between the β-Ga_2_O_3_ channel and AlN substrate. (**F**) XRD scan characteristics of β-Ga_2_O_3_ on AlN. a.u., arbitrary units. (**G**) High-resolution rocking curve of transferred and bonded β-Ga_2_O_3_ with an FWHM of 80 arc sec.

Figure 1A illustrates the heterogeneous integration process developed in this work. The monoclinic β-Ga_2_O_3_ is the most thermally stable phase of Ga_2_O_3_, and the single-crystal β-Ga_2_O_3_ substrate are available up to 6-inch in the industry (*31*). β-Ga_2_O_3_ has a large lattice constant of 12.2 Å along the (100) orientation, allowing the β-Ga_2_O_3_ thin film to be easily cleaved and transferred on the blue tape. Distinct from the planar exfoliation widely adopted in prior works, here we exfoliate Ga_2_O_3_ thin films from the edge of a 2-inch (-201) n-Ga_2_O_3_ substrate grown via the edge-defined film-fed growth (EFG). The substrate has an n-type doping concentration (*N*_D_) of 8 × 10^18^ cm^−3^ and a thickness of 500 μm. As the width of exfoliated film is determined by the substrate thickness, a consistent geometry around 5000 μm by 500 μm are produced in the repeated exfoliation. Subsequently, these exfoliated belts are placed in an array with a uniform belt-to-belt spacing, with the total array size scalable to the wafer level.

After the array formation, the β-Ga_2_O_3_ layer needs to be thinned down from tens of micrometers to below 500 nm for rf device fabrication. This is realized by multiple rounds of wafer-scale direct bonding at room temperature, which guarantees uniform bonding force across the wafer; the process details are elaborated in Materials and Methods. After reaching the target film thickness, β-Ga_2_O_3_ thin films are transferred from the carrier blue tape to a single-crystal 1-inch AlN substrate by a final bonding with a higher force and longer time. Figure 1B shows the optical microscopic images of a β-Ga_2_O_3_ belt transferred onto the AlN wafer. Figure 1C shows the thickness mapping of the 120 β-Ga_2_O_3_ belts across the wafer, revealing an average thickness of 428 nm with an SD of 123 nm. Photos of the key steps of this process are presented in fig. S1. Note that the Ga_2_O_3_ belt thickness uniformity may be further increased by the chemical-mechanical-planarization (CMP) process or choosing a thicker Ga_2_O_3_ crystal ingot, which can provide a more uniform Ga_2_O_3_ belt.

Table S1 of the Supplementary Materials compares this approach with three prior methods for heterogeneous integration—Scotch tape–based exfoliation, ion cutting, and fusion bonding. Here, the implantation-free, interfacial oxide-free approach with wafer-level scalability can potentially offer superior electrical and thermal properties for rf power devices. It can also be widely applicable to other van der Waals materials for heterogeneous integration. To showcase this applicability, we also fabricate Ga_2_O_3_ arrayed belts on the Si/SiO_2_ substrate and SiC substrate by the same process, which will be used as control samples in electrical and thermal characterizations.

As shown in Fig. 1D, the surface root mean square (RMS) roughness of β-Ga_2_O_3_ surface is 0.2 nm. Figure 1E shows the high-resolution transmission electron microscopy (HRTEM) image of the bonded AlN/Ga_2_O_3_ interface. The abrupt interface further confirms the absence of an interfacial dielectric layer. The Al, N, Ga, and O elements are confirmed by the energy-dispersive x-ray spectroscopy (EDX) mapping at the interface, as presented in fig. S2. As shown in Fig. 1F, x-ray diffraction (XRD) characterization of the β-Ga_2_O_3_ on AlN only reveals a sharp Ga_2_O_3_ peak and AlN peak, suggesting the absence of an interfacial material. Figure 1G shows the high-resolution rocking curve of the bonded β-Ga_2_O_3_ on AlN reveals a narrow full width at half maximum (FWHM) of 80 arc sec, verifying the high quality of the transferred β-Ga_2_O_3_.

### Device fabrication and thermal characterization

**Fig. 2.**
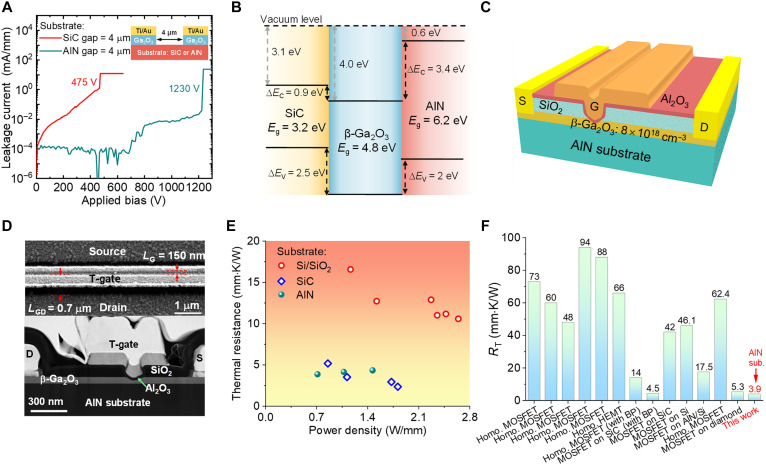
Electrical and thermal properties of the hetero-UWBG platform and device design. (**A**) Leakage current characteristics between two mesa structures fabricated on the β-Ga_2_O_3_ on the AlN sample and the β-Ga_2_O_3_ on the SiC sample, revealing a 2.5 times higher breakdown voltage in the β-Ga_2_O_3_ on the AlN sample. (**B**) Band alignment of SiC, β-Ga_2_O_3_, and AlN, where a large conduction band discontinuity of 3.4 eV is presented between β-Ga_2_O_3_ and AlN. (**C**) Cross-sectional schematic image of β-Ga_2_O_3_ rf power MOSFETs on the AlN substrate. (**D**) Top-view SEM and cross-sectional TEM images of a T-gate shaped β-Ga_2_O_3_ rf power MOSFET with *L*_G_ = 150 nm and a gated channel thickness of 30 nm. (**E**) Extracted thermal resistance (*R*_T_) versus dc power density of identical-geometry Ga_2_O_3_ MOSFETs fabricated on SiO_2_/Si, SiC, and AlN substrates; the same heterogeneous integration process is applied to three substrates. (**F**) *R*_T_ benchmark for this work and other reported Ga_2_O_3_ transistors on a variety of substrates. Data from refs. (*17*, *26*, *32*–*38*).

A mesa-shaped test device is first used to compare the voltage blocking capabilities of the fabricated Ga_2_O_3_-on-SiC and Ga_2_O_3_-on-AlN wafers (Fig. 2A). The *BV* between two electrodes with a spacing of 4 μm increases from 475 V in Ga_2_O_3_-on-SiC to 1230 V in Ga_2_O_3_-on-AlN, rendering a three times higher average electric field up to 3 MV/cm. This comparison illustrates the enhanced voltage blocking capabilities of the full-UWBG Ga_2_O_3_-on-AlN platform benefiting from the high *E*_C_ of the AlN substrate.

In addition to a higher breakdown field, the AlN substrate also enables superior electron confinement in the Ga_2_O_3_ channel. Figure 2B compares the band alignment diagram of the Ga_2_O_3_/SiC and Ga_2_O_3_/AlN heterojunctions. Benefiting from the low electron affinity of AlN, the Ga_2_O_3_/AlN junction provides a conduction band discontinuity of 3.4 eV, which is 2.5 eV higher than the Ga_2_O_3_/SiC junction. This high-potential back barrier can effectively suppress the channel carrier spill over and enhance the gate control over channel electrostatics. Such an enhanced gate control further allows for downscaling the gate length and increasing the channel doping, which improves *f*_T_/*f*_max_ and on-state current, respectively.

Figure 2C shows the three-dimensional schematic of the T-gate rf transistor fabricated on the Ga_2_O_3_-on-AlN wafer. A high *N*_D_ of 8 × 10^18^ cm^−3^, which is the *N*_D_ of the n^+^-Ga_2_O_3_ substrate in the host wafer, is adopted for the n-Ga_2_O_3_ channel. A recessed T-gate reduces the channel thickness below the gate, which suppresses the short-channel effect (SCE), and forms a field plate structure in the gate-to-drain access region, which enhances the device *BV*. Top-view and cross-sectional scanning electron microscopy (SEM) images of the fabricated β-Ga_2_O_3_ rf power metal oxide semiconductor field-effect transistor (MOSFET) are displayed as Fig. 2D, with the fabrication process detailed in the Materials and Methods section. Devices with various gate length (*L*_G_) and gate-to-drain distance (*L*_GD_) are fabricated. Devices with a *L*_G_/*L*_GD_/*L*_GS_ = 300/700/400 nm are used for rf power, noise, and linearity characterizations, whereas devices with a further scaled *L*_G_ = 150 nm and *L*_GD_ = 700 nm are used for the microwave noise measurements. Additional devices with a long *L*_G_ of 1 and 30 μm are used for thermal resistance (*R*_T_) and channel mobility measurements, respectively. From the transfer characteristic of the long-channel device, the electron mobility and density in the recessed Ga_2_O_3_ channel is extracted to be ~80 cm^2^/Vs and 1 × 10^13^ ~ 1.9 × 10^13^ cm^−2^, respectively, as shown in fig. S3.

The *R*_T_ of the fabricated Ga_2_O_3_-on-AlN, Ga_2_O_3_-on-Si/SiO_2_ and Ga_2_O_3_-on-SiC devices are measured by a transient thermal reflectance method, with the details described in the Materials and Methods section. As shown in Fig. 2E, the *R*_T_ of the Ga_2_O_3_-on-AlN device is measured to be 3.9 mm·K/W across a wide range of *P*_out_, which is similar to the *R*_T_ of Ga_2_O_3_-on-SiC device and over three times lower than that of the Ga_2_O_3_-on-Si/SiO_2_ device. Compared with other homogeneous or heterogeneously integrated Ga_2_O_3_ transistors reported in the literature (*17*, *26*, *32*–*38*), our Ga_2_O_3_-on-AlN device demonstrates one of the lowest *R*_T_, as shown in Fig. 2F. This performance is ascribed to the ultrathin Ga_2_O_3_ layer, high-*k*_T_ substrate, and the absence of the low-*k*_T_ interfacial bonding layer.

### Device characterization

**Fig. 3.**
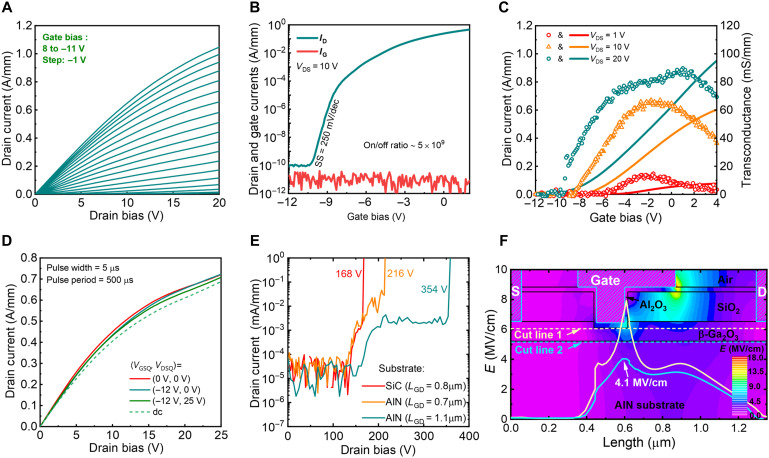
dc characteristics of the Ga_2_O_3_-on-AlN MOSFET. (**A**) Linear-scale *I*_D_-*V*_DS_ of a representative Ga_2_O_3_-on-AlN MOSFET with *L*_G_ = 300 nm. (**B**) Log-scale *I*_D_-*V*_GS_-*I*_G_ characteristics at *V*_DS_ = 10 V and (**C**) linear-scale *I*_D_-*V*_GS_-*g*_m_ characteristics at *V*_DS_ = 1/10/20 V of the same device. (**D**) Pulsed *I*_D_-*V*_DS_ characteristics of the device under three quiescent bias conditions with 5-μs pulse width, 1% duty cycle, and *V*_GS_ = 0 V. (**E**) Three-terminal off-state leakage current and breakdown characteristics of β-Ga_2_O_3_ MOSFETs fabricated on SiC and AlN substrates. (**F**) Simulated electric field contour within the Ga_2_O_3_-on-AlN MOSFET structure at a device breakdown voltage of 216 V, as well as the electric field profiles along the two cutlines in the Ga_2_O_3_ channel and AlN substrate. Although the breakdown occurs in Ga_2_O_3_, a high field of 4.1 MV/cm is present in the AlN substrate; if such a substrate field cannot be withstood by the substrate material, premature breakdown will occur in the substrate.

Figure 3A shows the output characteristics (*I*_D_-*V*_DS_) of a representative Ga_2_O_3_-on-AlN rf power MOSFET, revealing a maximum *I*_D_ of 1.05 A/mm. Log-scale transfer characteristic (*I*_D_-*V*_GS_-*I*_G_) at *V*_DS_ = 10 V is displayed in Fig. 3B, showing a subthreshold slope (SS) of 250 mV/dec and high on/off ratio of 5 × 10^9^. Linear-scale *I*_D_-*V*_GS_-*g*_m_ (transconductance) characteristics are shown in Fig. 3C at a *V*_DS_ of 1/10/20 V, revealing a maximum *g*_m,max_ of 90 mS/mm extracted at *V*_DS_ = 20 V. Benefited from the excellent gate control and channel electron confinement (fig. S4), the *g*_m_ remains 80% of the peak value across a wide *V*_GS_ range of 8.7 V (i.e., *V*_GS_ from −5.1 to 3.6 V), suggesting a high linearity.

Pulse *I*-*V* measurement is widely used to characterize device dynamic characteristics and identify possible degradation induced by carrier trapping. Figure 3D presents the gate-lag and drain-lag pulse *I*_D_-*V*_DS_ measurements at three different quiescent bias points (*V*_GSQ_, *V*_DSQ_) = (0 V, 0 V), (−12 V, 0 V), and (−12 V, 25 V), which represent the cold channel, gate pulse, and drain pulse scenarios, respectively. These measurements are performed at a pulse period of 5 μs, duty cycle of 1%, and *V*_GS_ = 0 V. Compared with dc characteristics, the pulsed characteristics under three conditions show nearly no dispersion up to a high *V*_DS_ = 25 V, validating the minimal carrier trapping in the device structure and the suppression of the SHE (standard hydrogen electrode). As a comparison, a nearly identical Ga_2_O_3_ device is fabricated on a Ga_2_O_3_-on-SiC wafer that is produced by the H-implanted ion cutting (*15*), which is found to suffer from considerable current collapse in pulse *I*-*V* measurement, with the details illustrated in fig. S5. This comparison manifests the advantage of the implantation-free integration process on achieving superior device dynamic characteristics.

Three-terminal off-state breakdown characteristics of β-Ga_2_O_3_ rf power MOSFETs fabricated on SiC and AlN substrates are shown in Fig. 3E. Ga_2_O_3_-on-AlN devices achieve a *BV* of 216 and 354 V for a *L*_GD_ = 0.7 and 1.1 μm, respectively, rendering an averaged breakdown field (*E*_ave_) of ~3 MV/cm. The faster increase in off-state drain current observed between *V*_DS_ = 150 and 200 V is most likely attributed to field-enhanced leakage mediated by carrier traps within the heavily doped Ga_2_O_3_ and at the AlN/Ga_2_O_3_ interface. Under a certain high voltage bias, the intense electric field in this region promotes emission from those traps via mechanisms such as Poole-Frenkel conduction, leading to increased leakage before catastrophic breakdown. A similar trap-mediated current hump has also been reported in power devices based on other materials such as gallium nitride (*39*). Further minimization of these defects includes piranha surface treatments to repair the bonded interface and annealing treatments to heal the heavily doped Ga_2_O_3_. In contrast, Ga_2_O_3_-on-SiC devices only achieve a *BV* = 168 V at a *L*_GD_ = 0.8 μm, corresponding to an *E*_ave_ of 2 MV/cm. The enhanced *BV* is further explained by physics-based technology computer-aided design (TCAD) simulations. The simulation models are described in the Materials and Methods section. The simulated electric field contour and extracted field profile along two cutlines are presented in Fig. 3F and fig. S6 for Ga_2_O_3_-on-AlN and Ga_2_O_3_-on-SiC devices, respectively. The simulation reveals that the *BV* of Ga_2_O_3_-on-SiC device is limited by the peak electric field in the SiC substrate. In Ga_2_O_3_-on-AlN devices, the substrate electric field is much lower than the *E*_C_ of AlN, thereby eliminating the premature substrate breakdown and allowing device to exploit the high *E*_C_ of Ga_2_O_3_ in the channel region.

**Fig. 4.**
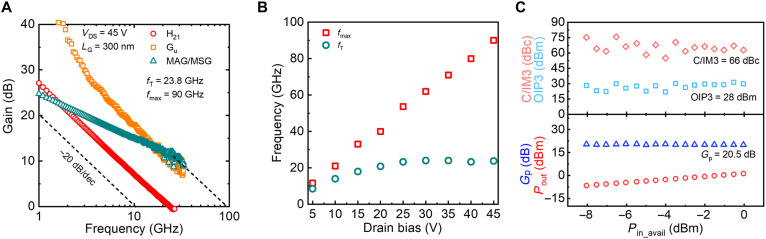
Small-signal rf characteristics and linearity characteristics of the Ga_2_O_3_-on-AlN MOSFET. (**A**) Small-signal gain characteristics of a Ga_2_O_3_-on-AlN MOSFET with *L*_G_ = 300 nm when biased at *V*_DS_ = 45 V and at the *V*_GS_ corresponding to the peak *g*_m_ and (**B**) extracted *f*_T_ and *f*_max_ versus *V*_DS_. A *f*_max_ = 90 GHz is extracted. (**C**) Two-tone load-pull *P*_in_ sweeps at *f* = 2 GHz to study the linearity of a device at *V*_DS_ = 15 V. A high linearity with OIP3 = 28 dBm is extracted.

Small-signal rf characteristics of β-Ga_2_O_3_ rf MOSFET with *L*_G_ = 300 nm is shown in Fig. 4A, when the device is biased at *V*_DS_ = 45 V and *V*_GS_ = 0 V. Standard off-wafer line-reflect-reflect-match (LRRM) calibration and pad-only parasitic from an on-wafer open structure are used to de-embed the as measured S-parameters. The *f*_T_ is determined to be 23.8 GHz, and a *f*_max_ of 90 GHz is determined by a conservative −20 dB/dec extrapolation slope from the maximum available gain/maximum stable gain (MAG/MSG). The *f*_T_ × *V*_DS_ is determined to be ~1 THz·V. The *f*_T_/*f*_max_ dependence on the *V*_DS_ is displayed in Fig. 4B. The *f*_T_ increases with *V*_DS_ and saturates at *V*_DS_ = 25~30 V, which is ascribed to the acceleration of electron velocity by electric field and the subsequent saturation. The *f*_max_ also increases with *V*_DS_ due to the reduced gate to drain capacitance because of the enhanced depletion. This can be understood by the analytical model *f*_max_ = 0.5*f*_T_/(2π*f*_T_ × *R*_g_ × *C*_gd_ + *R*_g_ × *g*_ds_)^0.5^, where *C*_gd_ and *g*_ds_ are gates to drain depletion capacitance and output conductance, respectively (*40*). The enhanced *f*_T_/*f*_max_ at high *V*_DS_ benefits the amplifiers in achieving a higher gain. The *f*_T_/*f*_max_ dependence on *V*_GS_ is summarized in fig. S7. Because of the flat *g*_m_, both *f*_T_ and *f*_max_ show weak dependence on *V*_GS_, suggesting a wide gate drive window for high-frequency operation.

The microwave noise performance of β-Ga_2_O_3_ MOSFET with *L*_G_ = 300 nm is summarized in section S9 of the Supplementary Materials. The minimal noise figure (*NF*_min_) is extracted to be ~1.1 dB at *f* = 8 GHz. By further scaling the *L*_G_ to 150 nm at a *V*_DS_ = 20 V, the *NF*_min_ is reduced to ~0.48 dB at *f* = 8 GHz, as shown in fig. S8. This *NF*_min_ reduction is ascribed to the higher carrier velocity underneath the gate and much lower *L*_G_, which can be understood by the relation *NF*_min_ ~ *f* × (*I*_D_*L*_G_/*E*_C_)^0.5^ × (ν_sat_)^−1^ × (*R*_S_ + *R*_G_)^0.5^, with the relation derivation illustrated in section S10 of the Supplementary Materials, where the *R*_S_ and *R*_G_ are the source and gate resistance, respectively (*40*). The optimal noise reflection coefficient |Γ_opt_| and phase angle ∠Γ_opt_ are displayed in fig. S10. The |Γ_opt_| and ∠|Γ_opt_| are measured to be 0.8 and 24° at *f* = 8 GHz, respectively. The as-measured noise parameters for representative device are shown in fig. S11. The *NF*_min_ is found to decrease at increased *V*_DS_ due to higher *g*_m_ and electron velocity.

The linearity performance of a β-Ga_2_O_3_ rf power MOSFET at *V*_DS_ = 15 V is characterized by two-tone load-pull input power (*P*_in_) sweeps at *f* = 2 GHz. Figure 4C shows the measured output third-order intercept point (OIP3) and power gain (*G*_a_) versus the *P*_in_. Benefited from the flat *g*_m_ at high *V*_DS_, a high OIP3 of 28 dBm and *G*_a_ = 20.5 dB are extracted. This reports the linearity performance in rf power MOSFETs based on either UWBG semiconductors or oxide semiconductors.

**Fig. 5.**
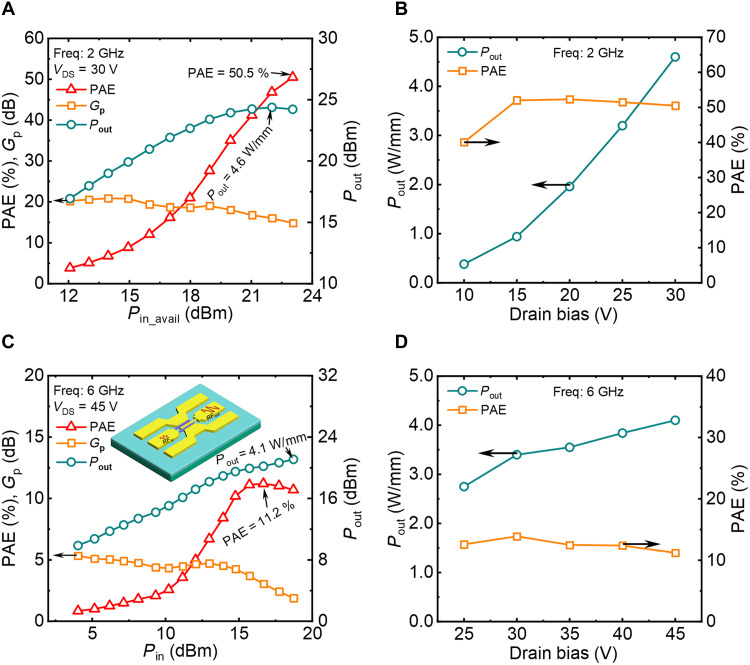
Large-signal rf characteristics of the Ga_2_O_3_-on-AlN MOSFET. (**A**) Large-signal performance of a Ga_2_O_3_-on-AlN MOSFET with *L*_GS_/*L*_GD_/*L*_G_ = 400/700/300 nm and *W*_G_ = 2 × 30 μm and the (**B**) extracted *P*_out_ and PAE dependence on *V*_DS_. The device is biased at class-AB condition with a frequency of 2 GHz, pulse width of 100 μs, and duty cycle of 10%. A record *P*_out_ = 4.6 W/mm at 2 GHz and PAE = 50.5% are achieved. (**C**) Large-signal class-AB performance of a Ga_2_O_3_-on-AlN MOSFET with *L*_GS_/*L*_GD_/*L*_G_ = 400/700/300 nm and *W*_G_ = 2 × 15 μm, with a power sweep at *f* = 6 GHz and *V*_DS_ = 45 V. Inset: Schematic of a Ga_2_O_3_-on-AlN MOSFET for an amplifier with rf signal amplification (**D**) *P*_out_ and PAE dependence on *V*_DS_ from 25 to 45 V at *f* = 6 GHz. A *P*_out_ = 4.1 W/mm at 6 GHz is extracted.

Figure 5A shows the large-signal and class-AB load-pull characteristics of the β-Ga_2_O_3_ rf MOSFET measured at *f* = 2 GHz and *V*_DS_ = 30 V. The input and output are matched, and the input signal is under pulse condition with a pulse width of 100 μs and duty cycle of 10%. By sweeping the *P*_in_ from 12 to 23 dBm, the *P*_out_ and PAE first increases and then saturates. Maximum *P*_out_ = 4.6 W/mm and PAE = 50.5% are achieved. The dependence of *P*_out_ and PAE on *V*_DS_ is summarized in Fig. 5B, revealing a *P*_out_ growing with *V*_DS_ with a relatively constant PAE. By further increasing *V*_DS_ to 50 V, the measured *P*_out_ remains saturated at around 4 W/mm. This *P*_out_ saturation is probably attributed to the current collapse under high *V*_DS_ bias induced by the traps located at the Ga_2_O_3_/AlN and Ga_2_O_3_/passivation layer interfaces. Further process refinement, such as piranha solution treatment and annealing that have been reported to be effective in interface trap minimization (*41*), could be explored to improve *P*_out_. Last, the C-band (6 GHz) load-pull measurement result of a device at *V*_DS_ = 45 V is displayed as Fig. 5C, revealing a *P*_out_ = 4.1 W/mm. Similar *P*_out_ and PAE versus *V*_DS_ trends at 6 GHz are identical to that of 2 GHz, as shown in Fig. 5D. These results demonstrate high *P*_out_ and PAE performance under a wide range of power and frequency in large-signal operation.

### Performance benchmarking and conclusion

**Fig. 6.**
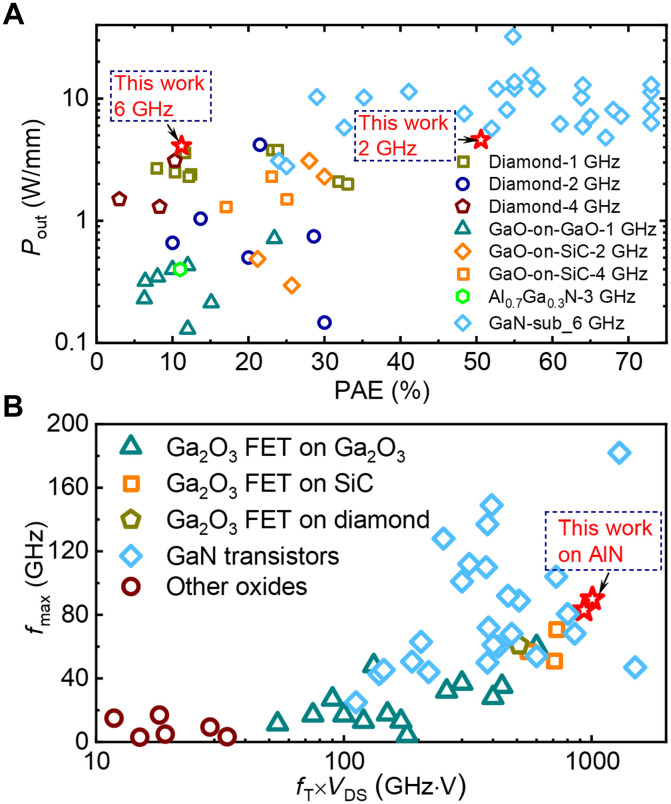
Benchmark of the rf metrics for the Ga_2_O_3_-on-AlN MOSFET against the state-of-the-art rf transistors. (**A**) *P*_out_ versus PAE of rf transistors reported in various UWBG material platforms, including diamond, Ga_2_O_3_-on-Ga_2_O_3_, Ga_2_O_3_-on-SiC, Al_0.7_Ga_0.3_N, and representative GaN rf transistors, reported at a frequency from 1 to 6 GHz. Data from refs. (*6*, *13*, *14*, *18*, *19*, *42**–**59*). (**B**) *f*_max_ versus *f*_T_ × *V*_DS_ of the best data reported in rf transistors based on various oxide materials, including Ga_2_O_3_, In_2_O_3_, ITO, IGZO, and IZO, and representative GaN rf transistors. Data from refs. (*6*, *11**–**14*, *18*, *19*, *42**–**45*, *57*, *58*, *61**–**73*). The device with *L*_G_/*W*_G_/*L*_GD_ = 0.3/2 × 30/0.7 μm has *f*_T_ = 23.3/23.8 GHz at *V*_DS_ = 40/45 V and *BV* = 216 V.

Among the UWBG rf power transistors reported in β-Ga_2_O_3_ (*6*, *13*, *14*, *18*, *19*, *42**–**45*), high Al-composition Al*_x_*Ga_1−*x*_N (*x* ≥ 50%) (*46*), and diamond (*47**–**53*), the Ga_2_O_3_-on-AlN transistor fabricated in this work presents the highest *P*_out_ and PAE (Fig. 6A). At 2 GHz, the *P*_out_ and PAE is six- and threefold higher than the best reports in Ga_2_O_3_ transistors on the native substrate (*13*), respectively, as well as 1.5- and twofold higher than Ga_2_O_3_-on-SiC devices (*19*, *45*). The *P*_out_ advantage over Ga_2_O_3_-on-SiC devices (*19*, *45*) expands to 2~4 times at the higher frequency of 4~6 GHz. It should be noted that the *P*_out_ performance of Ga_2_O_3_ rf transistors still lags behind that of GaN high-electron-mobility transistors (HEMTs) (*54**–**59*), primarily due to the still-maturing material and device process technologies considering the early stage of development. Whereas the introduction of heterogeneous integration has enabled substantial advances in the Ga_2_O_3_ rf FET, *P*_out_ improvement from 0.8 to over 4 W/mm with PAE increased from <20 to 50%. In addition, despite an inferior *P*_out_, the Ga_2_O_3_ rf FET has shown a higher average electric field than that in GaN HEMTs (usually around 1 MV/cm). Moving forward, we believe that the rf performance of hetero-UWBG devices can be further advanced along two pathways: (i) interface engineering to suppress the interfacial dielectric layer formation and reduce interfacial trap density and (ii) device optimizations to downscale gate, reduce ohmic contact resistance, and adopt multidimensional architectures such as FinFETs and multichannel designs (*60*).

In addition to UWBG semiconductors, β-Ga_2_O_3_ devices also belong to another device category—oxide rf transistors, which are widely used in back-end-of-line (BEOL) and small-signal applications (*61**–**66*). Here, we benchmark the small-signal performance of our device with representative GaN rf transistors (*57*, *58*, *67**–**71*) and the state-of-the-art oxide rf transistors reported in β-Ga_2_O_3_ (*6*, *11**–**14*, *18*, *19*, *42**–**45*, *72*, *73*), In_2_O_3_ (*61*, *62*), ITO (indium tin oxide) (*63*, *64*), IGZO (indium gallium zinc oxide) (*65*), and IZO (indium zinc oxide) (*66*) for the trade-off between *f*_max_ and *f*_T_ × *V*_DS_, as shown in Fig. 6B. Our Ga_2_O_3_-on-AlN rf transistor has reached a record-high *f*_T_ × *V*_DS_ = 1 THz·V and *f*_max_ = 90 GHz, respectively.

Last, to showcase the potential of Ga_2_O_3_-on-AlN rf MOSFET in not only high-power but also low-noise amplifiers, the device is benchmarked with state-of-the-art low-noise X-band transistors reported in GaAs, InP, GaN, and diamond, as shown in fig. S12. The *NF*_min_ = 0.48 dB is one of the lowest values among these X-band transistors, validating the high quality in materials and interfaces in the heterogeneously integrated Ga_2_O_3_-on-AlN structure.

In summary, this work presents a Ga_2_O_3_-on-AlN heterogeneously integrated platform for rf power devices, which can leverage the complementary electrical and thermal properties of multiple UWBG materials. The integration method combines exfoliated transfer and wafer-scale bonding, obviating the ion implantation and interfacial oxide required in many conventional methods, at the same time enabling a uniform geometry of the arrayed thin films with the total size scalable to the wafer level. The Ga_2_O_3_-on-AlN rf transistor leverages the high potential barrier offered by the AlN/Ga_2_O_3_ junction to realize tight gate control and gate length scalability in a highly doped Ga_2_O_3_ channel. In addition, the high *E*_C_ of the AlN substrate eliminates the premature substrate breakdown. These synergistic advances in the material, process, and device enable impressive frequency and power performance, including an average electric field over 3 MV/cm, a *f*_T_/*f*_max_ of 23.8/90 GHz, a *P*_out_ of 4.1 to 4.6 W/mm at 2 to 6 GHz, and a *NF*_min_ of 0.48 dB at 8 GHz, with the *P*_out_ being the highest in all UWBG rf devices and the noise among the one of the lowest in all representative X-band rf devices. These results suggest great potential of the heterogeneous UWBG platform for high-frequency, high-power, and low-noise rf electronics.

## MATERIALS AND METHODS

### Heterogeneous integration via exfoliation, layer transfer, and bonding

The (-201) β-Ga_2_O_3_ and Al-face AlN substrates are grown by edge-defined melt-grown and high-temperature high-pressure physical vapor transport (PVT) methods, respectively. The (-201) β-Ga_2_O_3_ (5 mm by 5 mm by 0.5 mm) and AlN (1 inch) substrates are both pretreated by piranha solution followed by buffered oxide etch (BOE) etch and water rinse. The β-Ga_2_O_3_ cleaved edge is manually bonded to the blue tape and forms an array. The initial thickness of the exfoliated β-Ga_2_O_3_ belts is ~10 μm. The thickness of the β-Ga_2_O_3_ belts or films on blue tape is reduced by multiple bonding processes as repeated peeling and re-bonding to fresh tape progressively remove sublayers through weak interfacial adhesion at cleavage planes. The ambient condition is high-purity nitrogen environment to avoid pollution. Thickness reduction per cycle (~20 to 30%) is monitored via cross-sectional SEM. When the thickness is reduced to ~500 nm, the blue tape is placed above a Si carrier wafer and graphite gasket with β-Ga_2_O_3_ side untouched. The AlN substrate is mounted at the top and then placed upside down into the bonding chamber. Room-temperature bonding with duration of 3 hours at a force of 3375 N is implemented by the SUSS MicroTec semiautomatic wafer bonder (SB6-8GEN2). The final thickness of the β-Ga_2_O_3_ films on the AlN substrate is around 400 nm.

### Fabrication of UWB Ga_2_O_3_-on-AlN rf power MOSFETs

Device full fabrication processes are summarized in fig. S13. After dry etching the β-Ga_2_O_3_ thin film, the final Ga_2_O_3_ thickness is reduced to ~60 nm and another piranha treatment is implemented to smooth the surface and partially repair the surface damage. Mesa isolation is realized by BCl_3_/Ar dry etching. The low-resistance alloyed ohmic contact is formed by evaporating Ti/Au (60/120 nm) followed by rapid thermal annealing at 450°C for 2 min. The contact resistance is measured to be 0.45 ohm·mm. A 100-nm SiO_2_ layer is deposited on the surface by plasma-enhanced chemical vapor deposition (PECVD) at 200°C. The channel recess region is formed by electron-beam lithography (EBL), which is followed by inductively coupled plasma reactive ion etching (ICP-RIE) of the SiO_2_ layer and ~30 nm of β-Ga_2_O_3_ to minimize the SCE and improve *f*_max_ as well as power gain. Another piranha treatment for 30 s is used to repair the etch damage and smoothen recess channel. The Al_2_O_3_ gate dielectric is formed by atomic layer deposition (ALD) with a thickness of 20 nm at a temperature of 300°C. The T-gate is formed by EBL and Ni/Au (50/350 nm) deposition and lift-off process. Source and drain ground-signal-ground pads were formed by etching the SiO_2_ layer. Identical Ga_2_O_3_ devices are fabricated on SiC and Si/SiO_2_ substrates for comparison.

### Device characterizations

The dc *I*-*V* and *C*-*V* characteristics were measured by the Keithley 4200 semiconductor analyzer systems and a Signatone probe station. Thermal resistance characterizations were performed by a transient thermal reflectance equipment with a 532-nm green light-emitting diode light source. The top surface of the gate electrode pad is gold as the thermoreflectance coefficient of gold at this wavelength is sufficiently high to enable accurate measurements. The thermoreflectance coefficient of gold was determined to be ~2.0 × 10^−4^ K^−1^, which is consistent with other reports (*74*, *75*). The schematic of a transient thermal reflectance setup was displayed in fig. S14. The S-parameter small-signal measurements were performed with an Agilent PNA-X N5245B network analyzer, and large signal measurements were performed with a Maury load pull system and Cascade probes. Microwave noise characterizations were performed using a Focus Microwaves noise measurement system. The two-tone linearity performance was evaluated using the Maury Microwave’s on-wafer load-pull measurement system, in which the two tones (*f*_1_, *f*_2_) were centered on 2 GHz (*f*_0_) with a tone spacing of 10 MHz. The linearity, noise, and load-pull characterizations setup were described in figs. S15 to S17.

### Device simulation models and parameters

In the TCAD simulation, the main physical models include the Lombardi model, Fermi-Dirac model, Shockley-Read-Hall model, Chynoweth model, and parallel electric field dependence model. The THERMCONTACT statement is adopted to specify the thermal boundary condition. The β-Ga_2_O_3_ impact ionization parameters of the Chynoweth model are based on the values reported in refs. (*76*, *77*). Details of these models are provided in the ATLAS user manual (*78*). Important simulation models and parameters are summarized in table S2 of the Supplementary Materials. Basic material parameters used in simulation are summarized in table S3 of the Supplementary Materials.

## Supplementary Materials

### This PDF file includes:

Supplementary Text Figs. S1 to S17 Tables S1 to S3 References

## References

[1] J. W. Palmour, S. T. Sheppard, R. P. Smith, S. T. Allen, W. L. Pribble, T. J. Smith, Z. Ring, J. J. Sumakeris, A. W. Saxler, J. W. Milligan, “Wide bandgap semiconductor devices and MMICs for RF power applications,” in *International Electron Devices Meeting. Technical Digest* (*Cat. No. 01CH37224*) (IEEE, 2001), pp. 14–17.

[2] J. Y. Tsao, S. Chowdhury, M. A. Hollis, D. Jena, N. M. Johnson, K. A. Jones, R. J. Kaplar, S. Rajan, C. G. Van de Walle, E. Bellotti, C. L. Chua, R. Collazo, M. E. Coltrin, J. A. Cooper, K. R. Evans, S. Graham, T. A. Grotjohn, E. R. Heller, M. Higashiwaki, M. S. Islam, P. W. Juodawlkis, M. A. Khan, A. D. Koehler, J. H. Leach, U. K. Mishra, R. J. Nemanich, R. C. N. Pilawa-Podgurski, J. B. Shealy, Z. Sitar, M. J. Tadjer, A. F. Witulski, M. Wraback, J. A. Simmons, Ultrawide-bandgap semiconductors: Research opportunities and challenges. Adv. Electron. Mater. 4, 1600501 (2018).

[3] Y. Zhang, A. Zubair, Z. Liu, M. Xiao, J. Perozek, Y. Ma, T. Palacios, GaN FinFETs and trigate devices for power and RF applications: Review and perspective. Semicond. Sci. Technol. 36, 054001 (2021).

[4] S. Pavlidis, G. Medwig, M. Thomas, Ultrawide-bandgap semiconductors for high-frequency devices. IEEE Microw. Mag. 25, 68–79 (2024).

[5] M. Higashiwaki, G. H. Jessen, Guest Editorial: The dawn of gallium oxide microelectronics. Appl. Phys. Lett. 112, 60401 (2018).

[6] N. Moser, K. Liddy, A. Islam, N. Miller, K. Leedy, T. Asel, S. Mou, A. Green, K. Chabak, Toward high voltage radio frequency devices in β-Ga_2_O_3_. Appl. Phys. Lett. 117, 242101 (2020).

[7] M. Porter, X. Yang, H. Gong, B. Wang, Z. Yang, Y. Zhang, Switching figure-of-merit, optimal design, and power loss limit of (ultra-) wide bandgap power devices: A perspective. Appl. Phys. Lett. 125, 110501 (2024).

[8] N. Donato, F. Udrea, Static and dynamic effects of the incomplete ionization in superjunction devices. IEEE Trans. Electron Devices 65, 4469–4475 (2018).

[9] T. Ohtsuki, T. Kamimura, M. Higashiwaki, Suppression of drain current leakage and short-channel effect in lateral Ga_2_O_3_ RF MOSFETs using (Al_x_Ga_1-x_)_2_O_3_ back-barrier. IEEE Electron Device Lett. 44, 1829–1832 (2023).

[10] X.-C. Wang, X.-L. Lu, Y.-L. He, F. Zhang, Y. Shao, P. Liu, Z.-N. Zhang, X.-F. Zheng, W.-W. Chen, L. Wang, J. Yang, X.-H. Ma, Y. Hao, Quasi-2D high mobility channel E-mode β-Ga_2_O_3_ MOSFET with Johnson FOM of 7.56 THz·V. Appl. Phys. Lett. 125, 63505 (2024).

[11] C. N. Saha, A. Vaidya, N. J. Nipu, L. Meng, D. S. Yu, H. Zhao, U. Singisetti, Thin channel Ga_2_O_3_ MOSFET with 55 GHz f_MAX_ and >100 V breakdown. Appl. Phys. Lett. 125, 62101 (2024).

[12] K. D. Chabak, D. E. Walker, A. J. Green, A. Crespo, M. Lindquist, K. Leedy, S. Tetlak, R. Gilbert, N. A. Moser, G. Jessen, “Sub-micron gallium oxide radio frequency field-effect transistors,” in *2018 IEEE MTT-S International Microwave Workshop Series on Advanced Materials and Processes for RF and THz Applications* (*IMWS-AMP*) (IEEE, 2018), pp. 1–3.

[13] N. A. Moser, T. Asel, K. J. Liddy, M. Lindquist, N. C. Miller, S. Mou, A. Neal, D. E. Walker, S. Tetlak, K. D. Leedy, G. H. Jessen, A. J. Green, K. D. Chabak, Pulsed power performance of β-Ga_2_O_3_ MOSFETs at L-band. IEEE Electron Device Lett. 41, 989–992 (2020).

[14] Y. Lv, H. Liu, Y. Wang, X. Fu, C. Ma, X. Song, X. Zhou, Y. Zhang, P. Dong, H. Du, S. Liang, T. Han, J. Zhang, Z. Feng, H. Zhou, S. Cai, Y. Hao, Oxygen annealing impact on β-Ga_2_O_3_ MOSFETs: Improved pinch-off characteristic and output power density. Appl. Phys. Lett. 117, 133503 (2020).

[15] W. Xu, Y. Wang, T. You, X. Ou, G. Han, H. Hu, S. Zhang, F. Mu, T. Suga, Y. Zhang, Y. Hao, X. Wang, “First demonstration of waferscale heterogeneous integration of Ga_2_O_3_ MOSFETs on SiC and Si substrates by ion-cutting process,” in *2019 IEEE International Electron Devices Meeting* (*IEDM*) (IEEE, 2019), pp. 12–15.

[16] W. Xu, T. Zhao, L. Zhang, K. Liu, H. Sun, Z. Qu, T. You, A. Yi, K. Huang, G. Han, F. Mu, T. Suga, X. Ou, Y. Hao, Thermal transport properties of β-Ga_2_O_3_ thin films on Si and SiC substrates fabricated by an ion-cutting process. ACS Appl. Electron. Mater. 6, 1710–1717 (2024).

[17] Y. Song, D. Shoemaker, J. H. Leach, C. M. Gray, H.-L. Huang, A. Bhattacharyya, Y. Zhang, C. U. Gonzalez-Valle, T. Hess, S. Zhukovsky, K. Ferri, R. M. Lavelle, C. Perez, D. W. Snyder, J.-P. Maria, B. Ramos-Alvarado, X. Wang, S. Krishnamoorthy, J. Hwang, B. M. Foley, S. Choi, Ga_2_O_3_-on-SiC composite wafer for thermal management of ultrawide bandgap electronics. ACS Appl. Mater. Interfaces 13, 40817–40829 (2021). 34470105 10.1021/acsami.1c09736

[18] X. Yu, W. Xu, Y. Wang, B. Qiao, R. Shen, J. Zhou, Z. Li, T. You, Z. Shen, K. Zhang, F.-F. Ren, D. Tang, X. Ou, G. Han, Y. Kong, T. Chen, S. Gu, Y. Zheng, J. Ye, R. Zhang, Heterointegrated Ga_2_O_3_-on-SiC RF MOSFETs with *f* _T_*/f*_max_ of 47/51 GHz by ion-cutting process. IEEE Electron Device Lett. 44, 1951–1954 (2023).

[19] M. Zhou, H. Zhou, S. Mengwei, G. Gao, X. Chen, X. Zhu, K. Dang, M. Peijun, M. Xiaohua, X. Zheng, Z. Liu, J. Zhang, Y. Zhang, Y. Hao, “71 GHz-f_max_ β-Ga_2_O_3_-on-SiC RF Power MOSFETs with Record P_out_= 3.1 W/mm and PAE= 50.8% at 2 GHz, P_out_= 2.3 W/mm at 4 GHz, and Low Microwave Noise Figure,” in *2024 IEEE Symposium on VLSI Technology and Circuits* (*VLSI Technology and Circuits*) (IEEE, 2024), pp. 1–2.

[20] J. Montes, C. Yang, H. Fu, T.-H. Yang, K. Fu, H. Chen, J. Zhou, X. Huang, Y. Zhao, Demonstration of mechanically exfoliated β-Ga_2_O_3_/GaN pn heterojunction. Appl. Phys. Lett. 114, 162103 (2019).

[21] Z. Cheng, L. Yates, J. Shi, M. J. Tadjer, K. D. Hobart, S. Graham, Thermal conductance across β-Ga_2_O_3_-diamond van der Waals heterogeneous interfaces. APL Mater. 7, 31118 (2019).

[22] J. Noh, S. Alajlouni, M. J. Tadjer, J. C. Culbertson, H. Bae, M. Si, H. Zhou, P. A. Bermel, A. Shakouri, P. D. Ye, High performance β-Ga_2_O_3_ nano-membrane field effect transistors on a high thermal conductivity diamond substrate. IEEE J. Electron Devices Soc. 7, 914–918 (2019).

[23] T. Matsumae, Y. Kurashima, H. Umezawa, K. Tanaka, T. Ito, H. Watanabe, H. Takagi, Low-temperature direct bonding of β-Ga_2_O_3_ and diamond substrates under atmospheric conditions. Appl. Phys. Lett. 116, 141602 (2020).

[24] Y. Zheng, E. Swinnich, J.-H. Seo, Investigation of thermal properties of β-Ga_2_O_3_ nanomembranes on diamond heterostructure using Raman thermometry. ECS J. Solid State Sci. Technol. 9, 055007 (2020).

[25] H. Zhou, K. Maize, J. Noh, A. Shakouri, P. D. Ye, Thermodynamic studies of β-Ga_2_O_3_ nanomembrane field-effect transistors on a sapphire substrate. ACS Omega 2, 7723–7729 (2017). 31457329 10.1021/acsomega.7b01313 PMC6645553

[26] Z. Qu, Y. Xie, T. Zhao, W. Xu, Y. He, Y. Xu, H. Sun, T. You, G. Han, Y. Hao, X. Ou, Extremely low thermal resistance of β-Ga_2_O_3_ MOSFETs by co-integrated design of substrate engineering and device packaging. ACS Appl. Mater. Interfaces 16, 57816–57823 (2024). 39388110 10.1021/acsami.4c08074 PMC11503635

[27] C. H. Lin, N. Hatta, K. Konishi, S. Watanabe, A. Kuramata, K. Yagi, M. Higashiwaki, Single-crystal-Ga_2_O_3_/polycrystalline-SiC bonded substrate with low thermal and electrical resistances at the heterointerface. Appl. Phys. Lett. 114, 32103 (2019).

[28] Y. Song, A. Bhattacharyya, A. Karim, D. Shoemaker, H.-L. Huang, S. Roy, C. M. Gray, J. H. Leach, J. Hwang, S. Krishnamoorthy, S. Choi, Ultra-wide band gap Ga_2_O_3_-on-SiC MOSFETs. ACS Appl. Mater. Interfaces 15, 7137–7147 (2023). 36700621 10.1021/acsami.2c21048

[29] C. Liu, Y. Wang, W. Xu, X. Jia, S. Huang, Y. Li, B. Li, Z. Luo, C. Fang, Y. Liu, T. You, X. Ou, Y. Hao, G. Han, Unique bias stress instability of heterogeneous Ga_2_O_3_-on-SiC MOSFET. IEEE Electron Device Lett. 44, 1256–1259 (2023).

[30] M. E. Levinshtein, S. L. Rumyantsev, M. S. Shur, *Properties of Advanced Semiconductor Materials: GaN*, *AIN*, *InN*, *BN*, *SiC*, *SiGe* (John Wiley & Sons, 2001).

[31] Novel Crystal Technology Inc., Novel Crystal Technology Achieves Breakthrough in Ga_2_O_3_ Crystal Growth, Paving Way for Larger, Higher-Quality Wafers, https://novelcrystal.co.jp/eng/2023/2340/.

[32] M. H. Wong, Y. Morikawa, K. Sasaki, A. Kuramata, S. Yamakoshi, M. Higashiwaki, Characterization of channel temperature in Ga_2_O_3_ metal-oxide-semiconductor field-effect transistors by electrical measurements and thermal modeling. Appl. Phys. Lett. 109, 193503 (2016).

[33] N. A. Blumenschein, N. A. Moser, E. R. Heller, N. C. Miller, A. J. Green, A. Popp, A. Crespo, K. Leedy, M. Lindquist, T. Moule, S. Dalcanale, E. Mercado, M. Singh, J. W. Pomeroy, M. Kuball, G. Wagner, T. Paskova, J. F. Muth, K. D. Chabak, G. H. Jessen, Self-heating characterization of β-Ga_2_O_3_ thin-channel MOSFETs by pulsed *I*–*V* and Raman nanothermography. IEEE Trans. Electron Devices 67, 204–211 (2020).

[34] D. Lei, K. Han, Y. Wu, Z. Liu, X. Gong, High performance Ga_2_O_3_ metal-oxide-semiconductor field-effect transistors on an AlN/Si substrate. IEEE J. Electron Devices Soc. 7, 596–600 (2019).

[35] T. Moule, M. Singh, S. Karboyan, E. Mercado, S. Dalcanale, M. J. Uren, Y. Zhang, “Electrical and thermal characterisation of β-(Al_x_Ga_(1-x)_)_2_O_3_/Ga_2_O_3_ HEMTs,” in *2019 International Conference on Compound Semiconductor Manufacturing Technology* (CS MANTECH, 2019).

[36] T. Zhao, X. Yu, W. Xu, Y. He, Z. Qu, R. Shen, R. Wang, H. Guo, H. Sun, Z. Li, M. Zhou, T. You, X. Ou, “First Demonstration of Wafer-Level Arrayed β-Ga_2_O_3_ Thin Films and MOSFETs on Diamond by Transfer Printing Technology,” in *2024 IEEE International Electron Devices Meeting* (*IEDM*) (IEEE, 2024), pp. 1–4.

[37] B. Chatterjee, K. Zeng, C. D. Nordquist, U. Singisetti, S. Choi, Device-level thermal management of gallium oxide field-effect transistors. IEEE Trans. Compon. Packag. Manuf. Technol. 9, 2352–2365 (2019).

[38] J. W. Pomeroy, C. Middleton, M. Singh, S. Dalcanale, M. J. Uren, M. H. Wong, K. Sasaki, A. Kuramata, S. Yamakoshi, M. Higashiwaki, M. Kuball, Raman thermography of peak channel temperature in β-Ga^2^O^3^ MOSFETs. IEEE Electron Device Lett. 40, 189–192 (2019).

[39] J. Liu, M. Xiao, R. Zhang, S. Pidaparthi, C. Drowley, L. Baubutr, A. Edwards, H. Cui, C. Coles, Y. Zhang, Trap-mediated avalanche in large-area 1.2 kV vertical GaN pn diodes. IEEE Electron Device Lett. 41, 1328–1331 (2020).

[40] J. W. Chung, W. E. Hoke, E. M. Chumbes, T. Palacios, AlGaN/GaN HEMT With 300-GHz *f*_max_. IEEE Electron Device Lett. 31, 195–197 (2010).

[41] H. Zhou, S. Alghmadi, M. Si, G. Qiu, P. D. Ye, Al_2_O_3_/β-Ga_2_O_3_ (-201) interface improvement through piranha pretreatment and post deposition annealing. IEEE Electron Device Lett. 37, 1411–1414 (2016).

[42] X. Yu, H. Gong, J. Zhou, Z. Shen, W. Xu, T. You, J. Wang, S. Zhang, Y. Wang, K. Zhang, R. Tao, Y. Wu, F.-F. Ren, X. Ou, Y. Kong, Z. Li, T. Chen, D. Chen, S. Gu, Y. Zheng, J. Ye, R. Zhang, High-voltage β-Ga_2_O_3_ RF MOSFETs with a shallowly-implanted 2DEG-like channel. IEEE Electron Device Lett. 44, 1060–1063 (2023).

[43] M. Singh, M. A. Casbon, M. J. Uren, J. W. Pomeroy, S. Dalcanale, S. Karboyan, P. J. Tasker, M. H. Wong, K. Sasaki, A. Kuramata, S. Yamakoshi, M. Higashiwaki, M. Kuball, Pulsed large signal RF performance of field-plated Ga_2_O_3_ MOSFETs. IEEE Electron Device Lett. 39, 1572–1575 (2018).

[44] A. J. Green, J. Speck, G. Xing, P. Moens, F. Allerstam, K. Gumaelius, T. Neyer, A. Arias-Purdue, V. Mehrotra, A. Kuramata, K. Sasaki, S. Watanabe, K. Koshi, J. Blevins, O. Bierwagen, S. Krishnamoorthy, K. Leedy, A. R. Arehart, A. T. Neal, S. Mou, S. A. Ringel, A. Kumar, A. Sharma, K. Ghosh, U. Singisetti, W. Li, K. Chabak, K. Liddy, A. Islam, S. Rajan, S. Graham, S. Choi, Z. Cheng, M. Higashiwaki, β-Gallium oxide power electronics. APL Mater. 10, 029201 (2022).

[45] M. Zhou, H. Zhou, S. Huang, M. Si, Y. Zhang, T. Luan, H. Yue, K. Dang, C. Wang, Z. Liu, J. Zhang, Y. Hao, “1.1 A/mm β-Ga_2_O_3_-on-SiC RF MOSFETs with 2.3 W/mm P_out_ and 30% PAE at 2 GHz and f_T_/f_max_ of 27.6/57 GHz,” in *2023 International Electron Devices Meeting* (*IEDM*) (IEEE, 2023), pp. 1–4.

[46] A. G. Baca, B. A. Klein, J. R. Wendt, S. M. Lepkowski, C. D. Nordquist, A. M. Armstrong, A. A. Allerman, E. A. Douglas, R. J. Kaplar, RF performance of Al_0.85_Ga_0.15_N/Al_0.70_Ga_0.30_N high electron mobility transistors with 80-nm gates. IEEE Electron Device Lett. 40, 17–20 (2019).

[47] T. G. Ivanov, J. Weil, P. B. Shah, A. G. Birdwell, K. Kingkeo, E. A. Viveiros, “Diamond RF Transistor Technology with f_t_= 41 GHz and f_max_= 44 GHz,” in *2018 IEEE/MTT-S International Microwave Symposium-IMS* (IEEE, 2018), pp. 1461–1463.

[48] S. Imanishi, K. Horikawa, N. Oi, S. Okubo, T. Kageura, A. Hiraiwa, H. Kawarada, 3.8 W/mm RF power density for ALD Al_2_O_3_-based two-dimensional hole gas diamond MOSFET operating at saturation velocity. IEEE Electron Device Lett. 40, 279–282 (2019).

[49] C. J. Zhou, J. J. Wang, J. C. Guo, C. Yu, Z. Z. He, Q. B. Liu, X. D. Gao, S. J. Cai, Z. H. Feng, Radiofrequency performance of hydrogenated diamond MOSFETs with alumina. Appl. Phys. Lett. 114, 063501 (2019).

[50] X. Yu, W. Hu, J. Zhou, B. Liu, T. Tao, Y. Kong, T. Chen, Y. Zheng, 1 W/mm output power density for H-terminated diamond MOSFETs with Al_2_O_3_/SiO_2_ bi-layer passivation at 2 GHz. IEEE J. Electron Devices Soc. 9, 160–164 (2021).

[51] K. Kudara, S. Imanishi, A. Hiraiwa, Y. Komatsuzaki, Y. Yamaguchi, Y. Kawamura, S. Shinjo, H. Kawarada, High output power density of 2DHG diamond MOSFETs with thick ALD-Al_2_O_3_. IEEE Trans. Electron Devices 68, 3942–3949 (2021).

[52] K. Kudara, M. Arai, Y. Suzuki, A. Morishita, J. Tsunoda, A. Hiraiwa, H. Kawarada, Over 1 A/mm drain current density and 3.6 W/mm output power density in 2DHG diamond MOSFETs with highly doped regrown source/drain. Carbon 188, 220–228 (2022).

[53] C. Yu, C. Zhou, J. Guo, Z. He, M. Ma, H. Yu, X. Song, A. Bu, Z. Feng, Hydrogen-terminated diamond MOSFETs on (0 0 1) single crystal diamond with state of the art high RF power density. Funct. Diam. 2, 64–70 (2022).

[54] Y. Wu, A. Saxler, M. Moore, R. P. Smith, S. Sheppard, P. M. Chavarkar, T. Wisleder, U. K. Mishra, P. Parikh, 30-W/mm GaN HEMTs by field plate optimization. IEEE Electron Device Lett. 25, 117–119 (2004).

[55] Y. Okamoto, Y. Ando, K. Hataya, T. Nakayama, H. Miyamoto, T. Inoue, M. Senda, K. Hirata, M. Kosaki, N. Shibata, M. Kuzuhara, Improved power performance for a recessed-gate AlGaN-GaN heterojunction FET with a field-modulating plate. IEEE Trans. Microw. Theory Tech. 52, 2536–2540 (2004).

[56] S. Kolluri, S. Keller, S. P. Denbaars, U. K. Mishra, N-polar GaN MIS-HEMTs with a 12.1-W/mm continuous-wave output power density at 4 GHz on sapphire substrate. IEEE Electron Device Lett. 32, 635–637 (2011).

[57] H. Lu, B. Hou, L. Yang, M. Zhang, L. Deng, M. Wu, Z. Si, S. Huang, X. Ma, Y. Hao, High RF performance GaN-on-Si HEMTs with passivation implanted termination. IEEE Electron Device Lett. 43, 188–191 (2021).

[58] S. Li, M. Wu, L. Yang, B. Yang, H. Sun, M. Zhang, B. Hou, H. Lu, X. Ma, Y. Hao, 15.1 W/mm power density GaN-on-GaN HEMT with high-gradient stepped-C doped buffer. IEEE Electron Device Lett. 46, 365–368 (2025).

[59] A. Bansal, R. Baby, A. Gowrisankar, V. S. Charan, R. Muralidharan, H. Chandrasekar, A. Sadhanala, S. Raghavan, D. N. Nath, Microwave power performance of buffer-free AlGaN/GaN MISHEMT with MOCVD grown ex situ SiN. IEEE Trans. Electron Devices 72, 2226–2232 (2025).

[60] Y. Zhang, F. Udrea, H. Wang, Multidimensional device architectures for efficient power electronics. Nat. Electron. 5, 723–734 (2022).

[61] A. Charnas, J. Anderson, J. Zhang, D. Zheng, D. Weinstein, P. D. Ye, Ultrathin indium oxide thin-film transistors with gigahertz operation frequency. IEEE Trans. Electron Devices 70, 532–536 (2023).

[62] D. Zheng, A. Charnas, J.-Y. Lin, J. Anderson, D. Weinstein, P. D. Ye, “Ultrathin Atomic-Layer-Deposited In_2_O_3_ Radio-Frequency Transistors with Record High f_T_ of 36 GHz and BEOL Compatibility,” in *2023 IEEE Symposium on VLSI Technology and Circuits* (*VLSI Technology and Circuits*) (IEEE, 2023), pp. 1–2.

[63] S. Li, M. Tian, Q. Gao, M. Wang, T. Li, Q. Hu, X. Li, Y. Wu, Nanometre-thin indium tin oxide for advanced high-performance electronics. Nat. Mater. 18, 1091–1097 (2019). 31406368 10.1038/s41563-019-0455-8

[64] Q. Hu, S. Zhu, C. Gu, S. Liu, M. Zeng, Y. Wu, Ultrashort 15-nm flexible radio frequency ITO transistors enduring mechanical and temperature stress. Sci. Adv. 8, eade4075 (2022). 36563154 10.1126/sciadv.ade4075 PMC9788755

[65] C. Tückmantel, U. Kalita, T. Haeger, M. Theisen, U. Pfeiffer, T. Riedl, Amorphous indium-gallium-zinc-oxide TFTs patterned by self-aligned photolithography overcoming the GHz threshold. IEEE Electron Device Lett. 41, 1786–1789 (2020).

[66] D. Zheng, A. Charnas, J. Anderson, H. Dou, Z. Hu, Z. Lin, Z. Zhang, J. Zhang, P.-Y. Liao, M. Si, H. Wang, D. Weinstein, P. D. Ye, “First demonstration of BEOL-compatible ultrathin atomiclayer-deposited InZnO transistors with GHz operation and record high bias-stress stability,” in *2022 International Electron Devices Meeting* (*IEDM*) (IEEE, 2022), pp. 3–4.

[67] J. Liu, Y. Zhou, J. Zhu, Y. Cai, K. M. Lau, K. J. Chen, DC and RF characteristics of AlGaN/GaN/InGaN/GaN double-heterojunction HEMTs. IEEE Trans. Electron Devices 54, 2–10 (2007).

[68] C.-W. Tsou, C.-Y. Lin, Y.-W. Lian, S. S. H. Hsu, 101-GHz InAlN/GaN HEMTs on silicon with high Johnson's figure-of-merit. IEEE Trans. Electron Devices 62, 2675–2678 (2015).

[69] S. Dai, Y. Zhou, Y. Zhong, K. Zhang, G. Zhu, H. Gao, Q. Sun, T. Chen, H. Yang, High *f_T_* AlGa (In) N/GaN HEMTs grown on Si with a low gate leakage and a high ON/OFF current ratio. IEEE Electron Device Lett. 39, 576–579 (2018).

[70] W. Song, Z. Zheng, T. Chen, J. Wei, L. Yuan, K. J. Chen, RF linearity enhancement of GaN-on-Si HEMTs with a closely coupled double-channel structure. IEEE Electron Device Lett. 42, 1116–1119 (2021).

[71] Q. Yu, C. Shi, L. Yang, H. Lu, M. Zhang, M. Wu, B. Hou, F. Jia, F. Guo, X. Ma, Y. Hao, High current and linearity AlGaN/GaN/-graded-AlGaN: Si-doped/GaN heterostructure for low voltage power amplifier application. IEEE Electron Device Lett. 44, 582–585 (2023).

[72] T. Kamimura, Y. Nakata, M. Higashiwaki, Delay-time analysis in radio-frequency β-Ga_2_O_3_ field effect transistors. Appl. Phys. Lett. 117, 253501 (2020).

[73] A. Vaidya, C. N. Saha, U. Singisetti, Enhancement mode β-(Al_x_Ga_1-x_)_2_O_3_/Ga_2_O_3_ heterostructure FET (HFET) with high transconductance and cutoff frequency. IEEE Electron Device Lett. 42, 1444–1447 (2021).

[74] N. Kumar, D. Vaca, C. Joishi, Z. Xia, S. Rajan, S. Kumar, Ultrafast thermoreflectance imaging and electrothermal modeling of β-Ga_2_O_3_ MESFETs. IEEE Electron Device Lett. 41, 641–644 (2020).

[75] T. Favaloro, J.-H. Bahk, A. Shakouri, Characterization of the temperature dependence of the thermoreflectance coefficient for conductive thin films. Rev. Sci. Instrum. 86, 024903 (2015). 25725873 10.1063/1.4907354

[76] K. Ghosh, U. Singisetti, Impact ionization in β-Ga_2_O_3_. J. Appl. Phys. 124, 085707 (2018).

[77] F. Zhou, H. Gong, M. Xiao, Y. Ma, Z. Wang, X. Yu, L. Li, L. Fu, H. H. Tan, Y. Yang, F.-F. Ren, S. Gu, Y. Zheng, H. Lu, R. Zhang, Y. Zhang, J. Ye, An avalanche-and-surge robust ultrawide-bandgap heterojunction for power electronics. Nat. Commun. 14, 4459 (2023). 37491528 10.1038/s41467-023-40194-0 PMC10368629

[78] Silvaco, Atlas User's Manual Device Simulation Software, http://silvaco.com.

[79] H. Fukui, Optimal noise figure of microwave GaAs MESFET's. IEEE Trans. Electron Devices 26, 1032–1037 (1979).

[80] D. Delagebeaudeuf, J. Chevrier, M. Laviron, P. Delescluse, A new relationship between the Fukui coefficient and optimal current value for low-noise operation of field-effect transistors. IEEE Electron Device Lett. 6, 444–445 (1985).

[81] S. Lardizabal, L. Dunleavy, W. Yau, S. Bar, “Experimental investigation of the temperature dependence of PHEMT noise parameters,” in *1994 IEEE MTT-S International Microwave Symposium Digest* (*Cat. No. 94CH3389-4*) (IEEE, 1994), pp. 845–848.

[82] W. Lu, V. Kumar, R. Schwindt, E. Piner, I. Adesida, DC, RF, and microwave noise performances of AlGaN/GaN HEMTs on sapphire substrates. IEEE Trans. Microw. Theory Tech. 50, 2499–2504 (2002).

[83] H. K. Huang, C. S. Wang, Y. Wang, C. L. Wu, C. S. Chang, Temperature effects of low noise InGaP/InGaAs/GaAs PHEMTs. Solid State Electron. 47, 1989–1994 (2003).

[84] J. Lee, A. Kuliev, V. Kumar, R. Schwindt, I. Adesida, Microwave noise characteristics of AlGaN/GaN HEMTs on SiC substrates for broad-band low-noise amplifiers. IEEE Microw. Wirel. Compon. Lett. 14, 259–261 (2004).

[85] W. Lu, J. Yang, M. A. Khan, I. Adesida, AlGaN/GaN HEMTs on SiC with over 100 GHz *f*_T_ and low microwave noise. IEEE Trans. Electron Devices 48, 581–585 (2001).

[86] H. Sun, A. R. Alt, H. Benedickter, C. R. Bolognesi, High-performance 0.1-μm gate AlGaN/GaN HEMTs on silicon with low-noise figure at 20 GHz. IEEE Electron Device Lett. 30, 107–109 (2009).

[87] J. S. Moon, D. Wong, P. Hashimoto, M. Hu, I. Milosavljevic, P. Willadsen, C. McGuire, S. Burnham, M. Micovic, M. Wetzel, D. Chow, Sub-1-dB noise figure performance of high-power field-plated GaN HEMTs. IEEE Electron Device Lett. 32, 297–299 (2010).

[88] Z. H. Liu, G. I. Ng, S. Arulkumaran, Y. K. T. Maung, K. L. Teo, S. C. Foo, V. Sahmuganathan, T. Xu, C. H. Lee, High microwave-noise performance of AlGaN/GaN MISHEMTs on silicon with Al_2_O_3_ gate insulator grown by ALD. IEEE Electron Device Lett. 31, 96–98 (2010).

[89] Z. H. Liu, G. I. Ng, S. Arulkumaran, Y. Maung, K. L. Teo, S. C. Foo, S. Vicknesh, Temperature-dependent microwave noise characteristics in ALD Al_2_O_3_/AlGaN/GaN MISHEMTs on silicon substrate. IEEE Electron Device Lett. 32, 318–320 (2011).

[90] T. Huang, O. Axelsson, T. N. T. Do, M. Thorsell, D. Kuylenstierna, N. Rorsman, Influence on noise performance of GaN HEMTs with in situ and low-pressure-chemical-vapor-deposition SiN*_x_* passivation. IEEE Trans. Electron Devices 63, 3887–3892 (2016).

[91] X. Liu, S. Zhang, K. Wei, J. Guo, X. He, Y. Zhang, H. Yin, S. Huang, X. Chen, Y. Zheng, X. Wang, S. Ouyang, Y. Li, 0.18 dB low-noise figure at 10 GHz for GaN MIS-HEMT with plasma-enhanced atomic layer deposition SiN layer. IEEE Electron Device Lett. 44, 1080–1083 (2023).

[92] A. Aleksov, A. Denisenko, U. Spitzberg, W. Ebert, E. Kohn, Microwave performance of diamond surface-channel FETs. IEEE Electron Device Lett. 23, 488–490 (2002).

[93] C. H. Lin, X. B. Mei, Y. C. Chou, L. S. Lee, J. M. Yang, M. Y. Nishimoto, P. H. Liu, R. To, A. Cavus, R. Tsai, M. Wojtowicz, R. Lai, “Sub-mW operation of InP HEMT X-band low-noise amplifiers for low power applications,” in *2009 Annual IEEE Compound Semiconductor Integrated Circuit Symposium* (IEEE, 2009), pp. 1–4.

[94] L. Liu, A. R. Alt, H. Benedickter, C. R. Bolognesi, InP-HEMT X-band low-noise amplifier with ultralow 0.6-mW power consumption. IEEE Electron Device Lett. 33, 209–211 (2011).

[95] D. C. Ruiz, T. Saranovac, D. Han, O. Ostinelli, C. R. Bolognesi, “Impact ionization control in 50 nm low-noise high-speed InP HEMTs with InAs channel insets,” in *2019 IEEE International Electron Devices Meeting* (*IEDM*) (IEEE, 2019), pp. 3–9.

