# Supplementary material for: Heterogeneous integration of ultrawide bandgap semiconductors for radio frequency power devices
Source: Sci Adv. 2025 Nov 19;11(47):eadw6167. doi: 10.1126/sciadv.adw6167 (PMC12629194; doi:10.1126/sciadv.adw6167)
Supplement: Supplementary file 1 — Supplementary Text Figs. S1 to S17 Tables S1 to S3 References [file sciadv.adw6167_sm.pdf]

Science Advances
AAAS

Supplementary Materials for

Heterogeneous integration of ultrawide bandgap semiconductors for radio frequency power devices

Hong Zhou *et al.*

Corresponding author: Min Zhou, minzhou@stu.xidian.edu.cn; Yuhao Zhang, yuhzhang@hku.hk; Jincheng Zhang, jchzhang@xidian.edu.cn

Sci. Adv. **11**, eadw6167 (2025)
DOI: 10.1126/sciadv.adw6167

This PDF file includes:

Supplementary Text
Figs. S1 to S17
Tables S1 to S3
References

Supplementary Text

1. Heterogenous integration process figures

A. Ga_2O_3 thinning via strong force bonding processes:

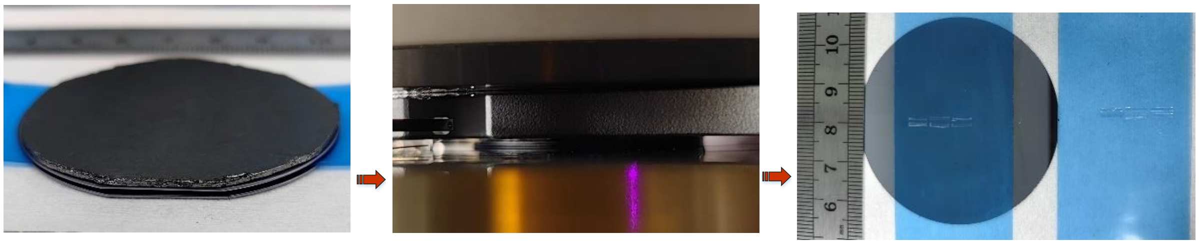

B. Microscope image of Ga_2O_3 on blue tape

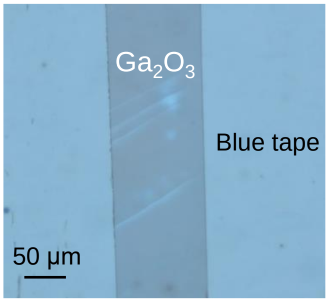

Fig. S1. The key steps of heterogenous integration process. (**A**) Zoomed-in view of the thinning of a few Ga_2O_3 belts via strong force bonding processes. (**B**) Top-view optical microscopic image of a Ga_2O_3 belt on blue tape after repeated bonding processes with the thickness reduced to ~500 nm.

2. Table S1. Comparison of the heterogenous integration technique developed in this work with other state-of-the-art heterogenous integration methods.

**Heterogenous Integration**	**Material damage**	**Area**	**Low-thermal-conductivity interfacial layer**
Ion-cutting Method	H-implantation induced damage	Wafer-scale integration	Yes
Scotch-tape Method	No	μm-size, random-sized flakes	No
Wafer-fusion Method	No	Wafer-scale integration	Yes
This Work	No	Wafer-scale integration	No

3. Al, N, Ga, O elements mapping at the heterojunction interface based on the energy dispersive X-ray (EDX)

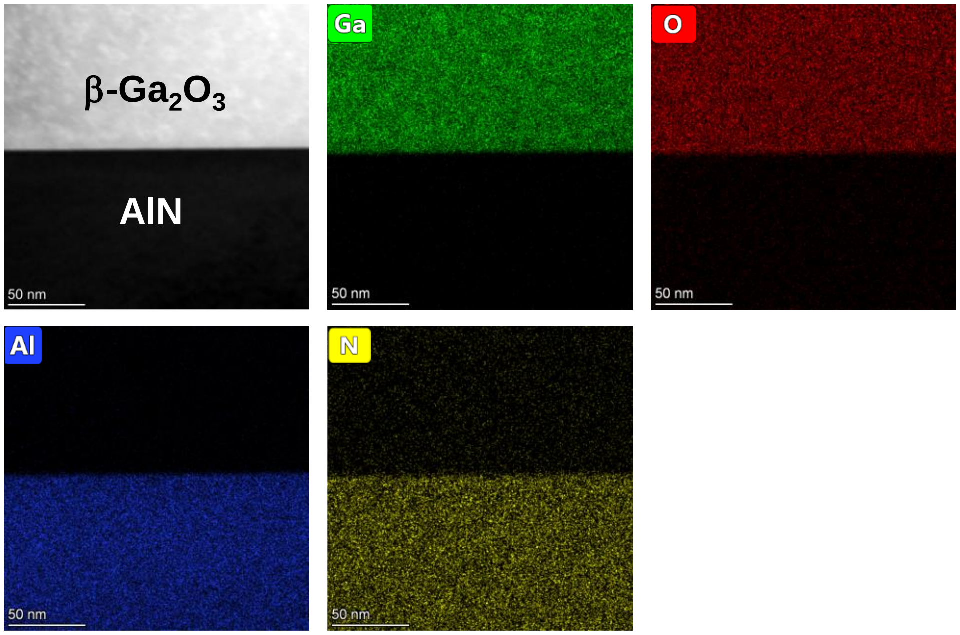

Fig. S2. EDX maps for Al, N, O, Ga elements at the heterogenous interface.

4. Electron concentration and mobility extraction

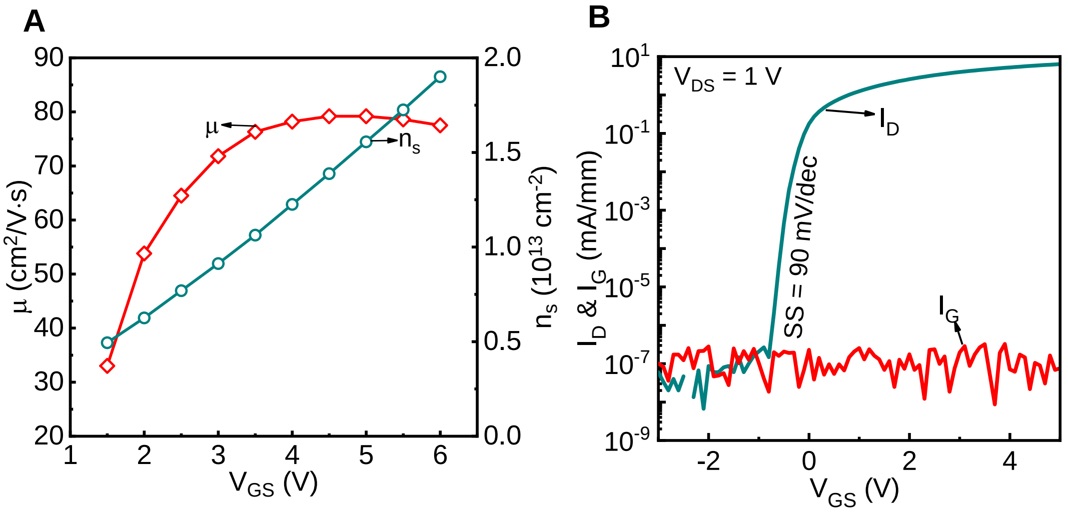

Fig. S3. Electron concentration and mobility extraction. (**A**) Extracted mobility and carrier density of a β-Ga_2O_3 on AlN MOSFET with a long L_G of 30 μm. (**B**) Transfer characteristics of the same device.

5. Transfer characteristics comparision between Ga_2O_3 MOSFETs on AlN and SiC substrate

At the same V_{DS} of 10 V, Ga_2O_3-on-AlN MOSFET shows a lower off-state leakage current, higher on/off ratio and lower subthreshold swing, indicating a better electron confinement at the pinch-off state, verifying the function of the AlN substrate for enhanced carrier confinement.

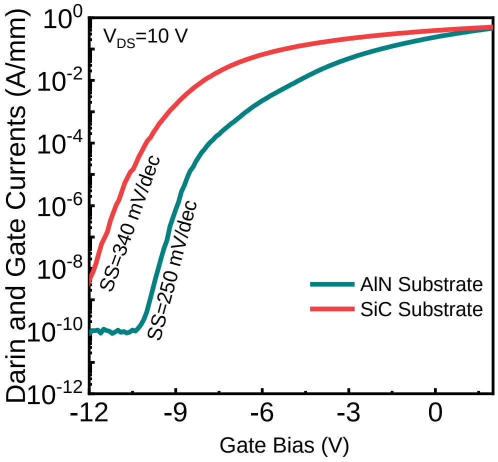

Fig. S4. Transfer characteristics comparison between Ga_2O_3 MOSFETs on AlN and SiC substrate.

6. Current collapse of device with H-implantation

The pulse I-V characteristics are measured at a 5 μs pulse width, 1% duty cycle and V_{GS} = 0 V. Gate pulsed and drain pulsed $I_{D,max}$ are found to be much lower than the DC $I_{D,max}$, showing the significant current-collapse effects due to the ion-implantation induced material damage. The ion-cutting process-based Ga_2O_3 MOSFET material fabrication is similar to previous reports (*15*, *16*) with H-ion implantation with a dose of 1×10^{17} cm^{-3} and energy of 35 keV. The device fabrication process, as well as the device structure and geometry, are identical to the primary device studied in this work.

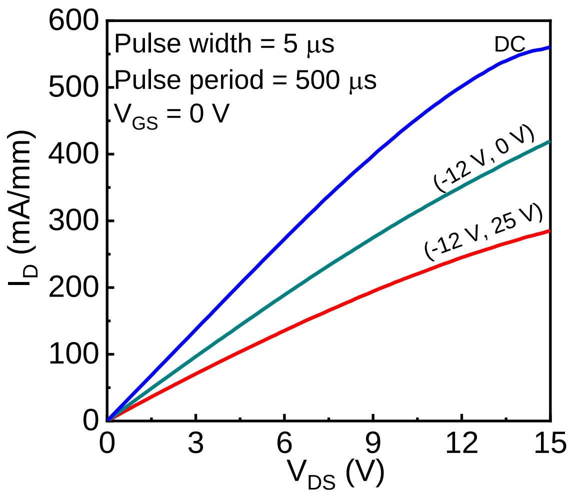

Fig. S5. Pulsed I_D-V_{DS} characteristics of the Ga_2O_3-on-AlN MOSFET fabricated based on the ion-cutting heterogenous integration process.

7. Ga_2O_3-on-SiC RF device electric field simulation

The peak electric field in the substrate exceeds the critical electric field of SiC, while the peak electric field in the channel is below the critical electric field of Ga_2O_3 (8 MV/cm), suggesting the premature breakdown occurs in the substrate.

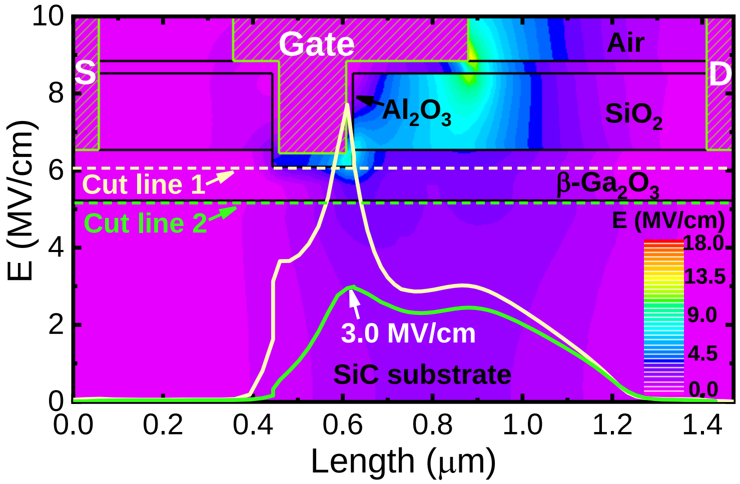

Fig. S6. Simulated electric field contour in a β-Ga_2O_3 MOSFET on SiC substrate at the device breakdown voltage of 168 V, as well as the extracted electric field profile along two cutlines in the Ga_2O_3 channel and the SiC substrate.

8. f_T/f_{max} dependence on V_{GS}

Due to the high linearity of the g_m, both f_T and f_{max} show a weak dependence on the V_{GS}.

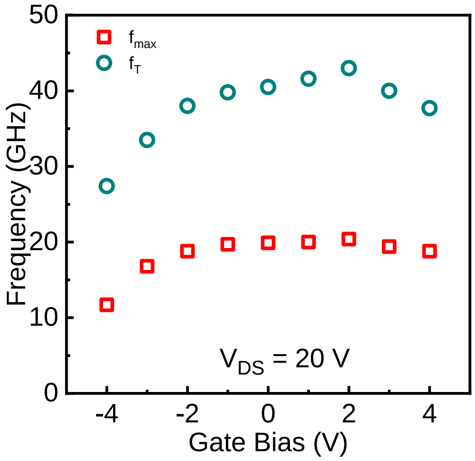

Fig. S7. f_T and f_{max} dependence on the V_{GS} at a fixed V_{DS} = 20 V.

9. Microwave noise properties of the Ga_2O_3-on-AlN MOSFET

A relatively low R_n is achieved, indicating that the β-Ga_2O_3-on-AlN material platform has high quality and low defect density.

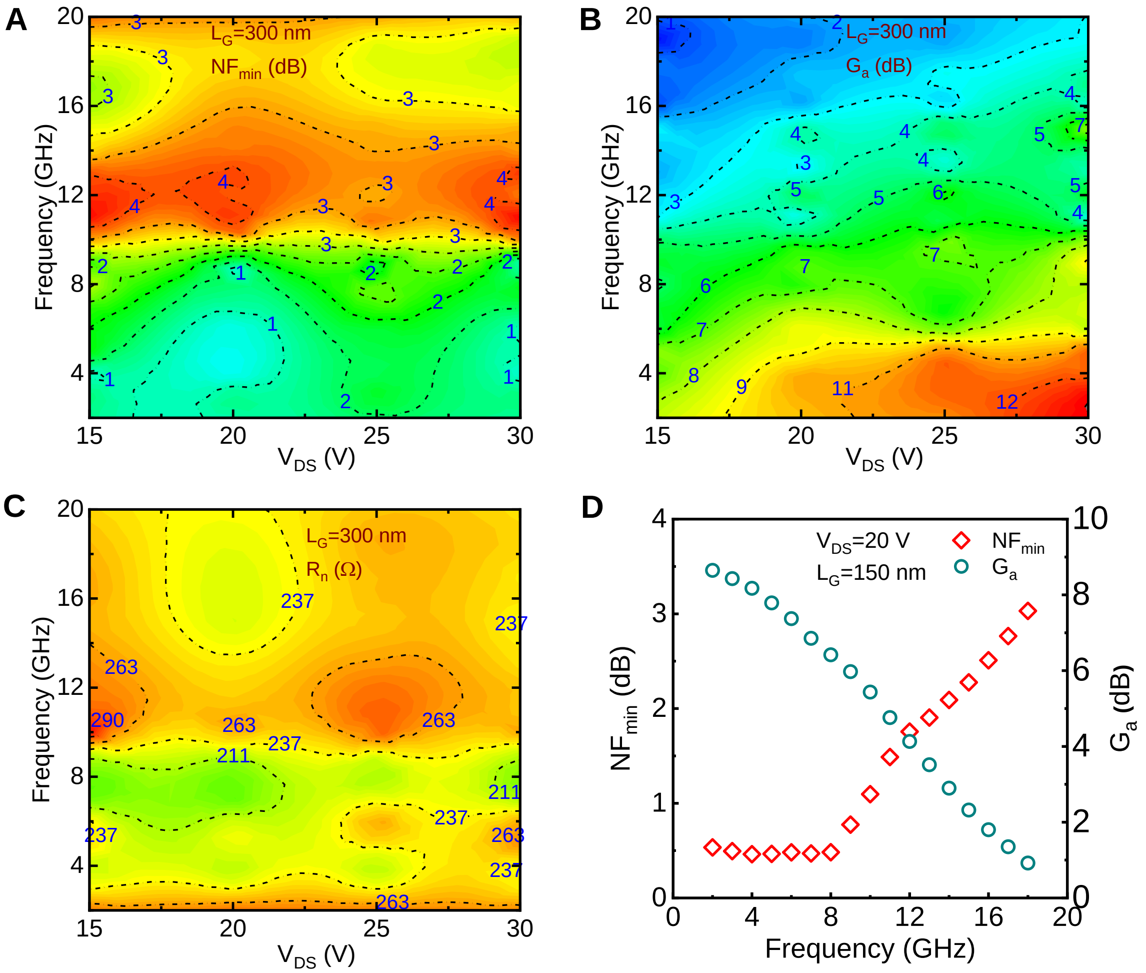

Fig. S8. Microwave noise characteristics of the Ga_2O_3-on-AlN MOSFET: Extracted microwave noise parameters (**A**) NF_{min}, (**B**) G_a and (**C**) R_n dependence on the V_{DS} and frequency in the V_{DS} range from 15 V to 30 V and frequency range from 2 GHz to 20 GHz. (**D**) Microwave noise characteristics of a device with a scaled L_G = 150 nm under the frequency from 2 GHz to 18 GHz at a fixed V_{DS} = 20 V.

10. Three more detailed explanations for the suitability of Ga_2O_3-on-AlN devices in low-noise RF applications

(1) High-quality Ga_2O_3 single crystal can be acquired via melt-grown method, exhibiting significantly lower dislocation densities and defects compared to materials grown using epitaxial techniques (e.g., GaN) on foreign substrates. Generally, a substantial portion of transistor noise originates from lattice defects in the channel material. Therefore, high-quality single-crystal material is the foundation to achieve a low-noise transistors. Additionally, the use of a high thermal conductivity substrate enhances the thermal dissipation of the Ga_2O_3 MOSFETs, further reducing thermal noise induced by junction temperature rise (self-heating effect).

(2) The suitability of Ga_2O_3 for low-noise applications can be further explained through the following formula (its derivations explained in the appendix afterwards):

$$NF_{\mathrm{min}} \approx 1+4\pi f \cdot \sqrt{\frac{I_{\mathrm{DS}} L_{\mathrm{G}}}{E_{\mathrm{C}}}} \cdot \frac{1}{v_{\mathrm{sat}}} \cdot \sqrt{(R_{\mathrm{S}}+R_{\mathrm{G}})} \tag{S1}$$

where NF_{min} is minimum-noise figure, f is the operating frequency, L_G is the gate length of transistors, E_C is the critical electric field of channel material, v_{sat} is the carrier saturation velocity, R_S and R_G are the source and gate series resistances, respectively. As the formula shows, the NF_{min} is inversely proportional to the bandgap energy and carrier saturation velocity. Ga_2O_3 possesses a bandgap energy far exceeding that of GaAs, and its electron saturation velocity (2×10^7 cm/s) is comparable that of GaAs. Therefore, Ga_2O_3 theoretically exhibits excellent potential for low-noise RF applications.

(3) An important component in the transmit/receiver (T/R) module for microwave communication systems is LNA. It determines the overall system noise figure. As shown in Fig. S9, a limiter is generally needed on the receive frontend to protect LNA against high input power. This limiter is typically implemented using discrete and expensive components. This limitation circuit degrades the noise performance and reduces the dynamic range of the front-end part. In addition, it also increases the complexity of the system design and raises the cost of the T/R module. The Ga_2O_3 LNA can sustain higher level of input power without limiter due to the ultra-wide bandgap of Ga_2O_3 material, as compared to the GaAs based LNA. Therefore, the T/R module with Ga_2O_3 LNA can eliminate the need for limiter and hence reduce the system complexity and the cost.

In conclusion, by integrating a high thermal conductivity substrate to mitigate the self-heating effects of Ga_2O_3 transistors, Ga_2O_3 exhibits significant potential for low-noise RF applications. This is attributed to its high critical electric field, high electron saturation velocity, and the availability of high-quality, large-size single-crystal materials, which collectively enhance device performance in microwave noise. As the first noise study of UWBG RF devices, we have demonstrated that the Ga_2O_3 RF transistors can achieve low NF_{min} comparable to that of GaN or GaAs transistors at X-Band. Ongoing advancements in material platform and device engineering (e.g., interface optimization, improvement of ohmic contacts) are expected to further improve the microwave-noise performance.

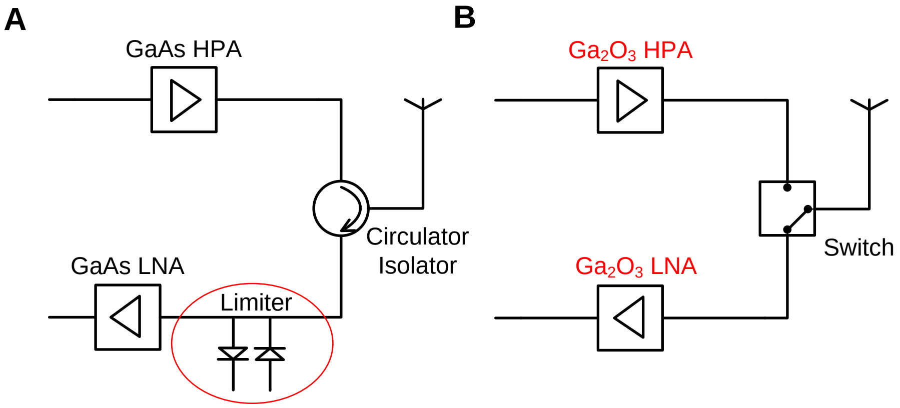

Fig. S9. Schematics of RF front end. (**A**) a classical GaAs front end and (**B**) a robust Ga_2O_3 front end.

Appendix:

The derivation process of the NF_{min} equation (S1):

According to the Fukui model, the NF_{min} can be expressed as (41):

$$NF_{min}=1+K_f\frac{f}{f_T}\cdot\sqrt{g_m(R_S+R_G)} \qquad (S2)$$

Where f_T is the cutoff frequency and g_m is the transconductance. The Fukui coefficient K_f is expressed as (42):

$$K_f=2\cdot\sqrt{\frac{I_{DS}}{E_C L_G g_m}} \qquad (S3)$$

The cutoff frequency f_T can be written as (43):

$$f_T=\frac{v_{sat}}{2\pi L_G} \qquad (S4)$$

Substituting Eq. (S3) and Eq. (S4) into Eq. (S2), we obtain

$$NF_{min}\sim f\cdot\sqrt{\frac{I_{DS}L_G}{E_C}}\cdot\frac{1}{v_{sat}}\cdot\sqrt{(R_S+R_G)}$$

11. Optimal noise reflection coefficient and phase angle dependence on frequency

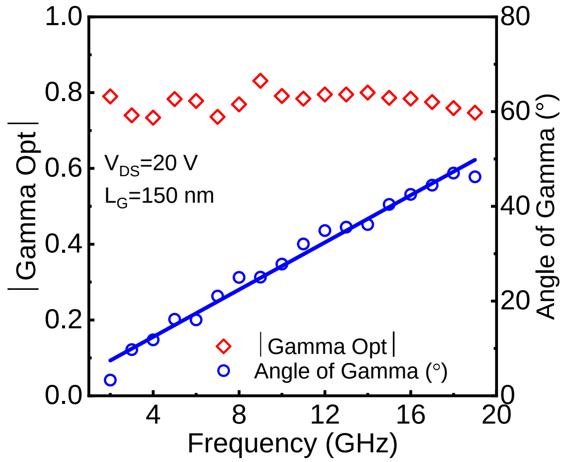

Fig. S10. The Optimal noise reflection coefficient $|\Gamma_{opt}|$ and phase angle $\angle\Gamma_{opt}$ versus frequency of a Ga_2O_3-on-AlN RF MOSFET with L_G = 150 nm.

12. As measured noise parameter figure

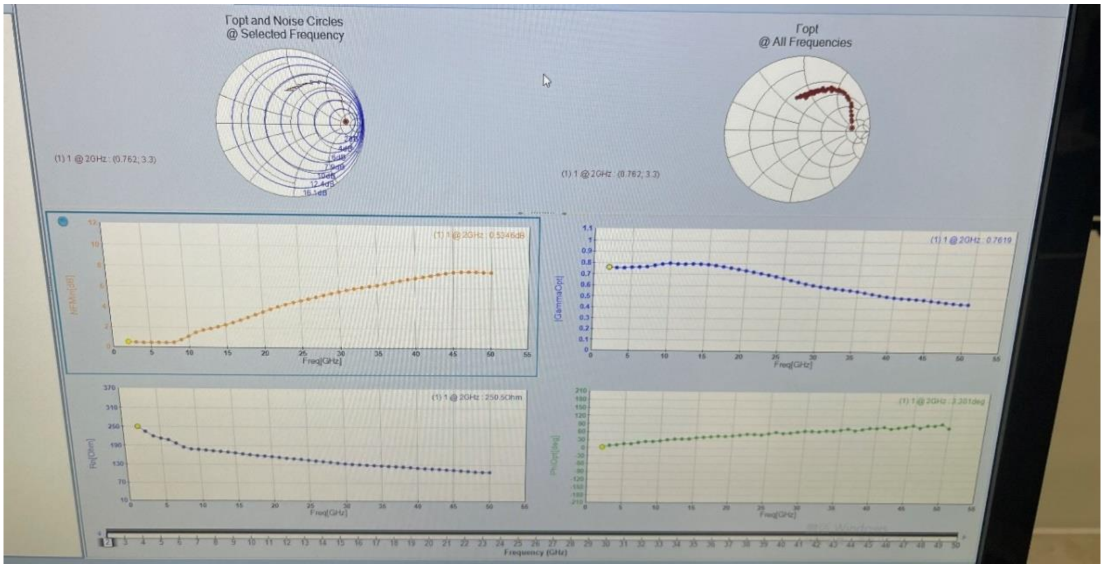

Fig. S11. As-measured noise parameters for representative Ga_2O_3 RF MOSFET on AlN substrate with gate length of 150 nm at a V_{DS} = 20 V, using a Focus microwave noise measurement system.

13. Benchmark of the microwave noise metric for the Ga_2O_3-on-AlN MOSFET against the state-of-the-art RF transistors

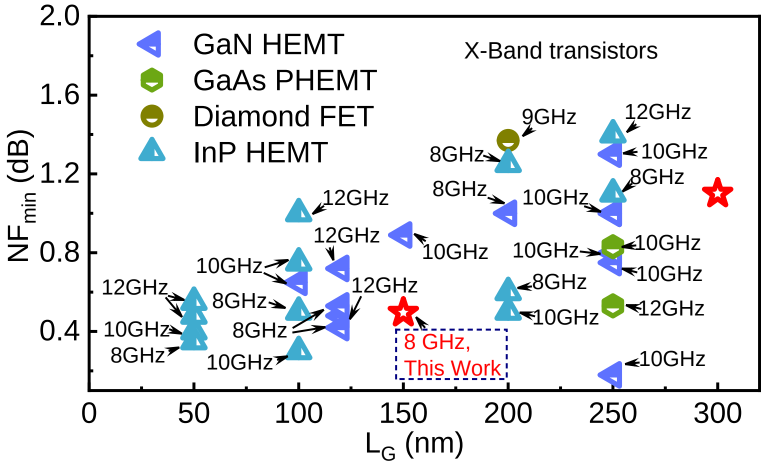

Fig. S12. ***NF*min vs. *L*G of the state-of-the-art low noise X-band RF transistors reported in diverse materials.** Data from references (*81*-*95*).

14. Device fabrication process flow

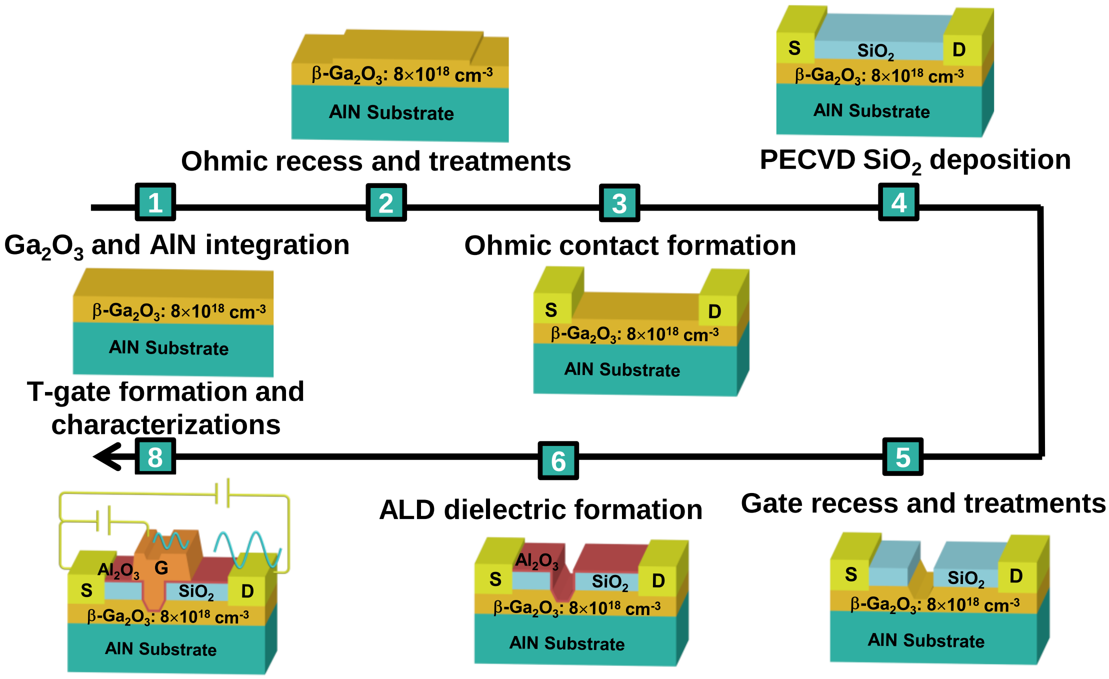

Fig. S13. Major fabrication steps of β-Ga_2O_3 RF FETs on AlN, including Ti/Au-based alloy contact formation, PECVD SiO_2 for field-plate oxide deposition, ALD Al_2O_3 gate dielectric and T-gate formation.

15. Transient thermal reflectance measurements schematic

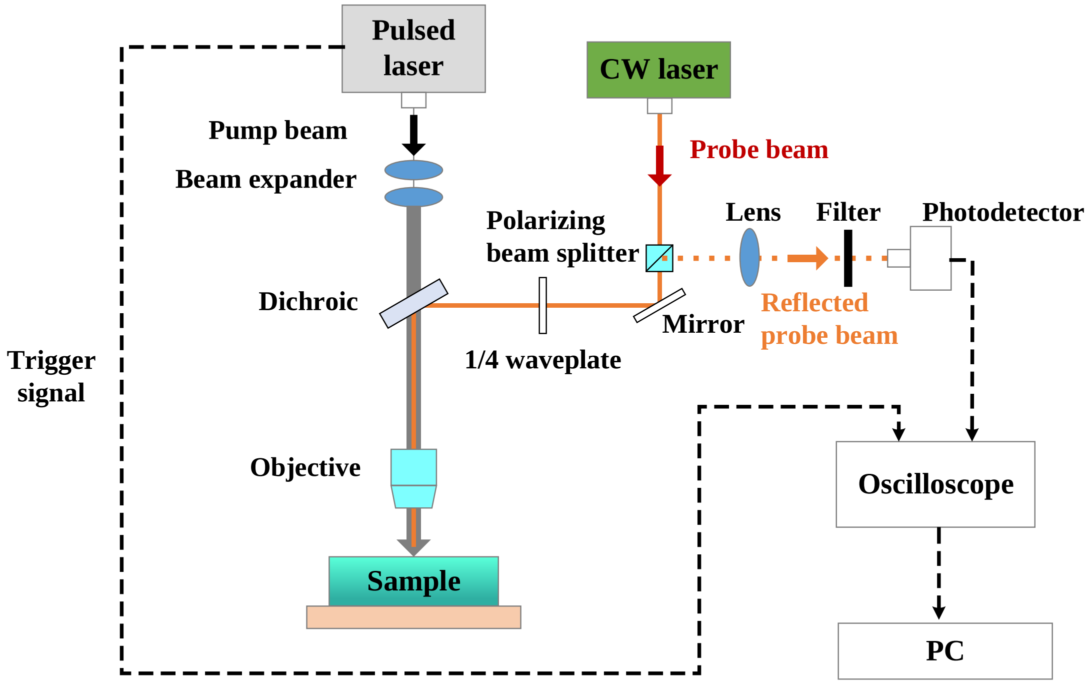

Fig. S14. Schematic of the transient thermal reflectance measurements set-up.

16. Linearity measurements schematic

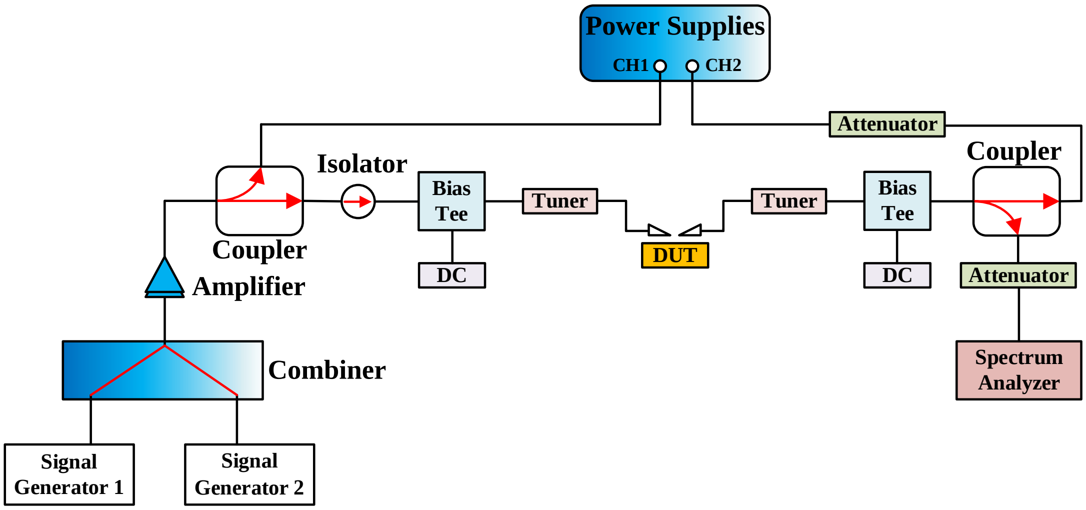

Fig. S15. Schematic of the two-tone linearity characterization set-up.

17. Microwave noise measurements schematic

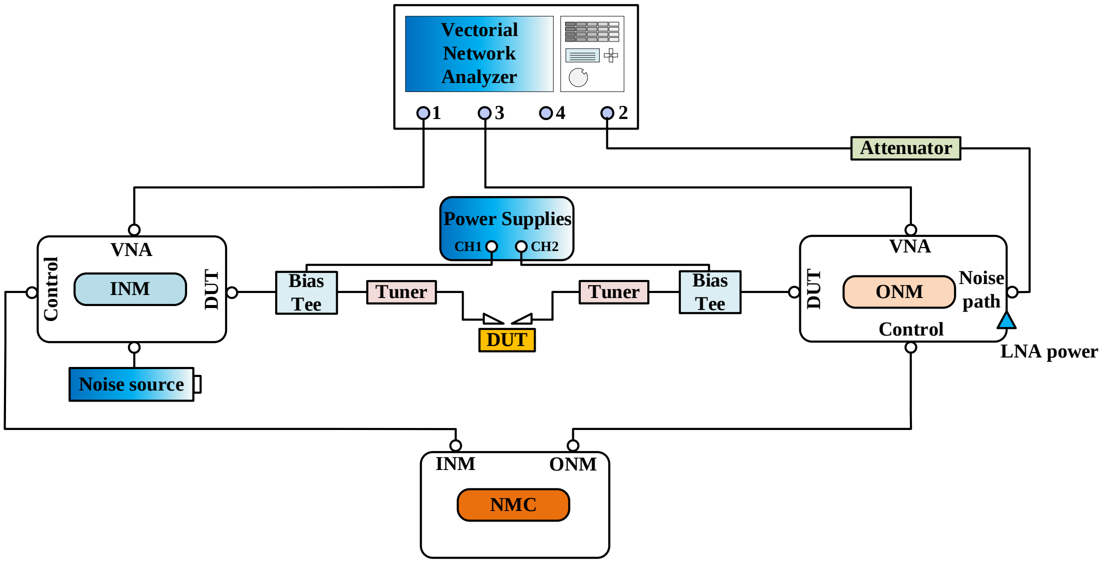

Fig. S16. Schematic of the microwave noise characterization set-up.

18. Load-pull measurements schematic

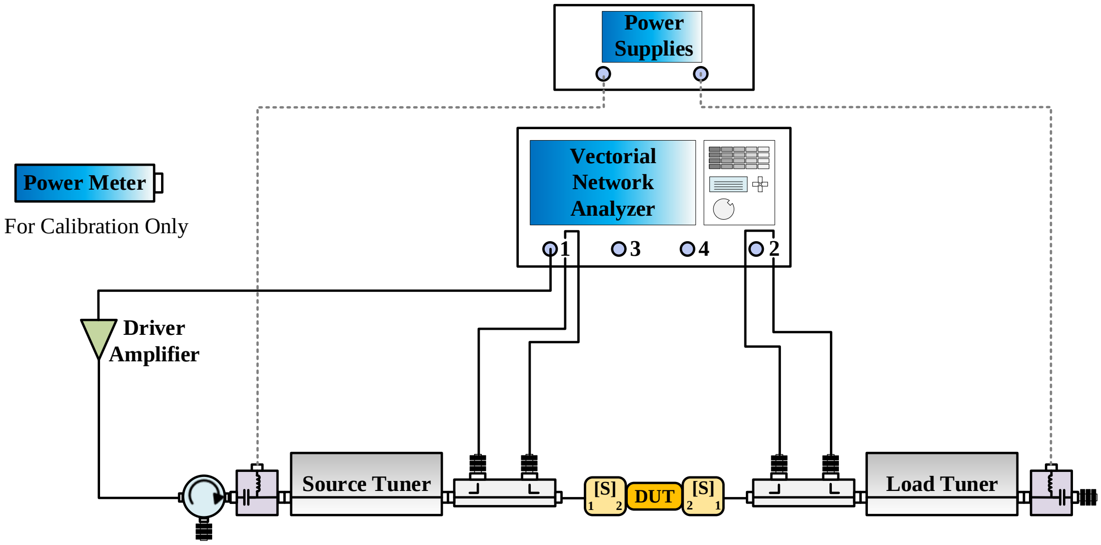

Fig. S17. Schematic of the large-signal load-pull characterization set-up.

19. Table S2. Key models and parameters in electric field contour simulation

where E is electric field, η is the fitting parameter, v_{sat} is the electron saturation velocity, $\mu_{n,0}$ is the low-field electron mobility, μ_n is the effective electron mobility, α_n and α_p are the impact ionization coefficient of electron and hole, respectively. A_n, B_n, A_p and B_p are constants related to the material.

**Physical Model**	**Model**	**Key parameters** (*7, 78-80*)
Mobility model	Parallel electric field dependence model $\mu_n = \frac{\mu_{n,0}}{\left[1+\left(\frac{\mu_{n,0}E}{v_{sat}}\right)^{\eta}\right]^{\frac{1}{\eta}}}$	$\eta = 1$<br>$v_{sat} = 2\times10^7$ cm/s<br>$\mu_{n,0} = 25$ cm^2/V·s
Impact ionization model	Chynoweth model $\alpha_n = A_n \times \exp\left(\frac{-B_n}{E}\right), \alpha_p = A_p \times \exp\left(\frac{-B_p}{E}\right)$	$A_n = 2.16\times10^6$ cm^{-1}<br>$B_n = 1.77\times10^7$ V/cm<br>$A_p = 5.75\times10^6$ cm^{-1}<br>$B_p = 1.77\times10^7$ V/cm

20. Table S3. Basic material parameters used in simulation

Material	Parameters
Ga_2O_3	Permittivity: 10<br>Band-gap: 4.8 eV<br>Affinity: 4.0 eV<br>Effective mass of electron: 0.28 m_0<br>Effective density of states<br>in the conduction band at 300 K: 3.72×10^{18} cm^{-3}
Al_2O_3	Permittivity: 8.9
4H-SiC	Permittivity: 9.7<br>Band-gap: 3.2 eV<br>Affinity: 3.1 eV
AlN	Permittivity: 9.76<br>Band-gap: 6.2 eV<br>Affinity: 0.6 eV
Ni	Work-function of gate metal: 5.12 eV

REFERENCES AND NOTES

1. J. W. Palmour, S. T. Sheppard, R. P. Smith, S. T. Allen, W. L. Pribble, T. J. Smith, Z. Ring, J. J. Sumakeris, A. W. Saxler, J. W. Milligan, "Wide bandgap semiconductor devices and MMICs for RF power applications," in *International Electron Devices Meeting. Technical Digest* (*Cat. No. 01CH37224*) (IEEE, 2001), pp. 14–17.

2. J. Y. Tsao, S. Chowdhury, M. A. Hollis, D. Jena, N. M. Johnson, K. A. Jones, R. J. Kaplar, S. Rajan, C. G. Van de Walle, E. Bellotti, C. L. Chua, R. Collazo, M. E. Coltrin, J. A. Cooper, K. R. Evans, S. Graham, T. A. Grotjohn, E. R. Heller, M. Higashiwaki, M. S. Islam, P. W. Juodawlkis, M. A. Khan, A. D. Koehler, J. H. Leach, U. K. Mishra, R. J. Nemanich, R. C. N. Pilawa-Podgurski, J. B. Shealy, Z. Sitar, M. J. Tadjer, A. F. Witulski, M. Wraback, J. A. Simmons, Ultrawide-bandgap semiconductors: Research opportunities and challenges. *Adv. Electron. Mater.* **4**, 1600501 (2018).

3. Y. Zhang, A. Zubair, Z. Liu, M. Xiao, J. Perozek, Y. Ma, T. Palacios, GaN FinFETs and trigate devices for power and RF applications: Review and perspective. *Semicond. Sci. Technol.* **36**, 054001 (2021).

4. S. Pavlidis, G. Medwig, M. Thomas, Ultrawide-bandgap semiconductors for high-frequency devices. *IEEE Microw. Mag.* **25**, 68–79 (2024).

5. M. Higashiwaki, G. H. Jessen, Guest Editorial: The dawn of gallium oxide microelectronics. *Appl. Phys. Lett.* **112**, 60401 (2018).

6. N. Moser, K. Liddy, A. Islam, N. Miller, K. Leedy, T. Asel, S. Mou, A. Green, K. Chabak, Toward high voltage radio frequency devices in β-Ga_2O_3. *Appl. Phys. Lett.* **117**, 242101 (2020).

7. M. Porter, X. Yang, H. Gong, B. Wang, Z. Yang, Y. Zhang, Switching figure-of-merit, optimal design, and power loss limit of (ultra-) wide bandgap power devices: A perspective. *Appl. Phys. Lett.* **125**, 110501 (2024).

8. N. Donato, F. Udrea, Static and dynamic effects of the incomplete ionization in superjunction devices. *IEEE Trans. Electron Devices* **65**, 4469–4475 (2018).

9. T. Ohtsuki, T. Kamimura, M. Higashiwaki, Suppression of drain current leakage and short-channel effect in lateral Ga_2O_3 RF MOSFETs using $(Al_xGa_{1-x})_2O_3$ back-barrier. *IEEE Electron Device Lett.* **44**, 1829–1832 (2023).

10. X.-C. Wang, X.-L. Lu, Y.-L. He, F. Zhang, Y. Shao, P. Liu, Z.-N. Zhang, X.-F. Zheng, W.-W. Chen, L. Wang, J. Yang, X.-H. Ma, Y. Hao, Quasi-2D high mobility channel E-mode β-Ga_2O_3 MOSFET with Johnson FOM of 7.56 THz·V. *Appl. Phys. Lett.* **125**, 63505 (2024).

11. C. N. Saha, A. Vaidya, N. J. Nipu, L. Meng, D. S. Yu, H. Zhao, U. Singisetti, Thin channel Ga_2O_3 MOSFET with 55 GHz f_{MAX} and >100 V breakdown. *Appl. Phys. Lett.* **125**, 62101 (2024).

12. K. D. Chabak, D. E. Walker, A. J. Green, A. Crespo, M. Lindquist, K. Leedy, S. Tetlak, R. Gilbert, N. A. Moser, G. Jessen, "Sub-micron gallium oxide radio frequency field-effect transistors," in *2018 IEEE MTT-S International Microwave Workshop Series on Advanced Materials and Processes for RF and THz Applications* (*IMWS-AMP*) (IEEE, 2018), pp. 1–3.

13. N. A. Moser, T. Asel, K. J. Liddy, M. Lindquist, N. C. Miller, S. Mou, A. Neal, D. E. Walker, S. Tetlak, K. D. Leedy, G. H. Jessen, A. J. Green, K. D. Chabak, Pulsed power performance of β-Ga_2O_3 MOSFETs at L-band. *IEEE Electron Device Lett.* **41**, 989–992 (2020).

14. Y. Lv, H. Liu, Y. Wang, X. Fu, C. Ma, X. Song, X. Zhou, Y. Zhang, P. Dong, H. Du, S. Liang, T. Han, J. Zhang, Z. Feng, H. Zhou, S. Cai, Y. Hao, Oxygen annealing impact on β-Ga_2O_3 MOSFETs: Improved pinch-off characteristic and output power density. *Appl. Phys. Lett.* **117**, 133503 (2020).

15. W. Xu, Y. Wang, T. You, X. Ou, G. Han, H. Hu, S. Zhang, F. Mu, T. Suga, Y. Zhang, Y. Hao, X. Wang, "First demonstration of waferscale heterogeneous integration of Ga_2O_3 MOSFETs on SiC and Si substrates by ion-cutting process," in *2019 IEEE International Electron Devices Meeting* (*IEDM*) (IEEE, 2019), pp. 12–15.

16. W. Xu, T. Zhao, L. Zhang, K. Liu, H. Sun, Z. Qu, T. You, A. Yi, K. Huang, G. Han, F. Mu, T. Suga, X. Ou, Y. Hao, Thermal transport properties of β-Ga_2O_3 thin films on Si and SiC

substrates fabricated by an ion-cutting process. *ACS Appl. Electron. Mater.* **6**, 1710–1717 (2024).

17. Y. Song, D. Shoemaker, J. H. Leach, C. M. Gray, H.-L. Huang, A. Bhattacharyya, Y. Zhang, C. U. Gonzalez-Valle, T. Hess, S. Zhukovsky, K. Ferri, R. M. Lavelle, C. Perez, D. W. Snyder, J.-P. Maria, B. Ramos-Alvarado, X. Wang, S. Krishnamoorthy, J. Hwang, B. M. Foley, S. Choi, Ga_2O_3-on-SiC composite wafer for thermal management of ultrawide bandgap electronics. *ACS Appl. Mater. Interfaces* **13**, 40817–40829 (2021).

18. X. Yu, W. Xu, Y. Wang, B. Qiao, R. Shen, J. Zhou, Z. Li, T. You, Z. Shen, K. Zhang, F.-F. Ren, D. Tang, X. Ou, G. Han, Y. Kong, T. Chen, S. Gu, Y. Zheng, J. Ye, R. Zhang, Heterointegrated Ga_2O_3-on-SiC RF MOSFETs with f_T/f_{max} of 47/51 GHz by ion-cutting process. *IEEE Electron Device Lett.* **44**, 1951–1954 (2023).

19. M. Zhou, H. Zhou, S. Mengwei, G. Gao, X. Chen, X. Zhu, K. Dang, M. Peijun, M. Xiaohua, X. Zheng, Z. Liu, J. Zhang, Y. Zhang, Y. Hao, "71 GHz-f_{max} β-Ga_2O_3-on-SiC RF Power MOSFETs with Record P_{out}= 3.1 W/mm and PAE= 50.8% at 2 GHz, P_{out}= 2.3 W/mm at 4 GHz, and Low Microwave Noise Figure," in *2024 IEEE Symposium on VLSI Technology and Circuits* (*VLSI Technology and Circuits*) (IEEE, 2024), pp. 1–2.

20. J. Montes, C. Yang, H. Fu, T.-H. Yang, K. Fu, H. Chen, J. Zhou, X. Huang, Y. Zhao, Demonstration of mechanically exfoliated β-Ga_2O_3/GaN pn heterojunction. *Appl. Phys. Lett.* **114**, 162103 (2019).

21. Z. Cheng, L. Yates, J. Shi, M. J. Tadjer, K. D. Hobart, S. Graham, Thermal conductance across β-Ga_2O_3-diamond van der Waals heterogeneous interfaces. *APL Mater.* **7**, 31118 (2019).

22. J. Noh, S. Alajlouni, M. J. Tadjer, J. C. Culbertson, H. Bae, M. Si, H. Zhou, P. A. Bermel, A. Shakouri, P. D. Ye, High performance β-Ga_2O_3 nano-membrane field effect transistors on a high thermal conductivity diamond substrate. *IEEE J. Electron Devices Soc.* **7**, 914–918 (2019).

23. T. Matsumae, Y. Kurashima, H. Umezawa, K. Tanaka, T. Ito, H. Watanabe, H. Takagi, Low-temperature direct bonding of β-Ga_2O_3 and diamond substrates under atmospheric conditions. *Appl. Phys. Lett.* **116**, 141602 (2020).

24. Y. Zheng, E. Swinnich, J.-H. Seo, Investigation of thermal properties of β-Ga_2O_3 nanomembranes on diamond heterostructure using Raman thermometry. *ECS J. Solid State Sci. Technol.* **9**, 055007 (2020).

25. H. Zhou, K. Maize, J. Noh, A. Shakouri, P. D. Ye, Thermodynamic studies of β-Ga_2O_3 nanomembrane field-effect transistors on a sapphire substrate. *ACS Omega* **2**, 7723–7729 (2017).

26. Z. Qu, Y. Xie, T. Zhao, W. Xu, Y. He, Y. Xu, H. Sun, T. You, G. Han, Y. Hao, X. Ou, Extremely low thermal resistance of β-Ga_2O_3 MOSFETs by co-integrated design of substrate engineering and device packaging. *ACS Appl. Mater. Interfaces* **16**, 57816–57823 (2024).

27. C. H. Lin, N. Hatta, K. Konishi, S. Watanabe, A. Kuramata, K. Yagi, M. Higashiwaki, Single-crystal-Ga_2O_3/polycrystalline-SiC bonded substrate with low thermal and electrical resistances at the heterointerface. *Appl. Phys. Lett.* **114**, 32103 (2019).

28. Y. Song, A. Bhattacharyya, A. Karim, D. Shoemaker, H.-L. Huang, S. Roy, C. M. Gray, J. H. Leach, J. Hwang, S. Krishnamoorthy, S. Choi, Ultra-wide band gap Ga_2O_3-on-SiC MOSFETs. *ACS Appl. Mater. Interfaces* **15**, 7137–7147 (2023).

29. C. Liu, Y. Wang, W. Xu, X. Jia, S. Huang, Y. Li, B. Li, Z. Luo, C. Fang, Y. Liu, T. You, X. Ou, Y. Hao, G. Han, Unique bias stress instability of heterogeneous Ga_2O_3-on-SiC MOSFET. *IEEE Electron Device Lett.* **44**, 1256–1259 (2023).

30. M. E. Levinshtein, S. L. Rumyantsev, M. S. Shur, *Properties of Advanced Semiconductor Materials: GaN, AIN, InN, BN, SiC, SiGe* (John Wiley & Sons, 2001).

31. Novel Crystal Technology Inc., Novel Crystal Technology Achieves Breakthrough in Ga_2O_3 Crystal Growth, Paving Way for Larger, Higher-Quality Wafers, https://novelcrystal.co.jp/eng/2023/2340/.

32. M. H. Wong, Y. Morikawa, K. Sasaki, A. Kuramata, S. Yamakoshi, M. Higashiwaki, Characterization of channel temperature in Ga_2O_3 metal-oxide-semiconductor field-effect transistors by electrical measurements and thermal modeling. *Appl. Phys. Lett.* **109**, 193503 (2016).

33. N. A. Blumenschein, N. A. Moser, E. R. Heller, N. C. Miller, A. J. Green, A. Popp, A. Crespo, K. Leedy, M. Lindquist, T. Moule, S. Dalcanale, E. Mercado, M. Singh, J. W. Pomeroy, M. Kuball, G. Wagner, T. Paskova, J. F. Muth, K. D. Chabak, G. H. Jessen, Self-heating characterization of β-Ga_2O_3 thin-channel MOSFETs by pulsed *I–V* and Raman nanothermography. *IEEE Trans. Electron Devices* **67**, 204–211 (2020).

34. D. Lei, K. Han, Y. Wu, Z. Liu, X. Gong, High performance Ga_2O_3 metal-oxide-semiconductor field-effect transistors on an AlN/Si substrate. *IEEE J. Electron Devices Soc.* **7**, 596–600 (2019).

35. T. Moule, M. Singh, S. Karboyan, E. Mercado, S. Dalcanale, M. J. Uren, Y. Zhang, "Electrical and thermal characterisation of β-$(Al_xGa_{(1-x)})_2O_3/Ga_2O_3$ HEMTs," in *2019 International Conference on Compound Semiconductor Manufacturing Technology* (CS MANTECH, 2019).

36. T. Zhao, X. Yu, W. Xu, Y. He, Z. Qu, R. Shen, R. Wang, H. Guo, H. Sun, Z. Li, M. Zhou, T. You, X. Ou, "First Demonstration of Wafer-Level Arrayed β-Ga_2O_3 Thin Films and MOSFETs on Diamond by Transfer Printing Technology," in *2024 IEEE International Electron Devices Meeting* (*IEDM*) (IEEE, 2024), pp. 1–4.

37. B. Chatterjee, K. Zeng, C. D. Nordquist, U. Singisetti, S. Choi, Device-level thermal management of gallium oxide field-effect transistors. *IEEE Trans. Compon. Packag. Manuf. Technol.* **9**, 2352–2365 (2019).

38. J. W. Pomeroy, C. Middleton, M. Singh, S. Dalcanale, M. J. Uren, M. H. Wong, K. Sasaki, A. Kuramata, S. Yamakoshi, M. Higashiwaki, M. Kuball, Raman thermography of peak channel temperature in β-Ga^2O^3 MOSFETs. *IEEE Electron Device Lett.* **40**, 189–192 (2019).

39. J. Liu, M. Xiao, R. Zhang, S. Pidaparthi, C. Drowley, L. Baubutr, A. Edwards, H. Cui, C. Coles, Y. Zhang, Trap-mediated avalanche in large-area 1.2 kV vertical GaN pn diodes. *IEEE Electron Device Lett.* **41**, 1328–1331 (2020).

40. J. W. Chung, W. E. Hoke, E. M. Chumbes, T. Palacios, AlGaN/GaN HEMT With 300-GHz f_{max}. *IEEE Electron Device Lett.* **31**, 195–197 (2010).

41. H. Zhou, S. Alghmadi, M. Si, G. Qiu, P. D. Ye, Al_2O_3/β-Ga_2O_3 (-201) interface improvement through piranha pretreatment and post deposition annealing. *IEEE Electron Device Lett.* **37**, 1411–1414 (2016).

42. X. Yu, H. Gong, J. Zhou, Z. Shen, W. Xu, T. You, J. Wang, S. Zhang, Y. Wang, K. Zhang, R. Tao, Y. Wu, F.-F. Ren, X. Ou, Y. Kong, Z. Li, T. Chen, D. Chen, S. Gu, Y. Zheng, J. Ye, R. Zhang, High-voltage β-Ga_2O_3 RF MOSFETs with a shallowly-implanted 2DEG-like channel. *IEEE Electron Device Lett.* **44**, 1060–1063 (2023).

43. M. Singh, M. A. Casbon, M. J. Uren, J. W. Pomeroy, S. Dalcanale, S. Karboyan, P. J. Tasker, M. H. Wong, K. Sasaki, A. Kuramata, S. Yamakoshi, M. Higashiwaki, M. Kuball, Pulsed large signal RF performance of field-plated Ga_2O_3 MOSFETs. *IEEE Electron Device Lett.* **39**, 1572–1575 (2018).

44. A. J. Green, J. Speck, G. Xing, P. Moens, F. Allerstam, K. Gumaelius, T. Neyer, A. Arias-Purdue, V. Mehrotra, A. Kuramata, K. Sasaki, S. Watanabe, K. Koshi, J. Blevins, O. Bierwagen, S. Krishnamoorthy, K. Leedy, A. R. Arehart, A. T. Neal, S. Mou, S. A. Ringel, A. Kumar, A. Sharma, K. Ghosh, U. Singisetti, W. Li, K. Chabak, K. Liddy, A. Islam, S. Rajan, S. Graham, S. Choi, Z. Cheng, M. Higashiwaki, β-Gallium oxide power electronics. *APL Mater.* **10**, 029201 (2022).

45. M. Zhou, H. Zhou, S. Huang, M. Si, Y. Zhang, T. Luan, H. Yue, K. Dang, C. Wang, Z. Liu, J. Zhang, Y. Hao, "1.1 A/mm β-Ga_2O_3-on-SiC RF MOSFETs with 2.3 W/mm P_{out} and 30% PAE at 2 GHz and f_T/f_{max} of 27.6/57 GHz," in *2023 International Electron Devices Meeting* (*IEDM*) (IEEE, 2023), pp. 1–4.

46. A. G. Baca, B. A. Klein, J. R. Wendt, S. M. Lepkowski, C. D. Nordquist, A. M. Armstrong, A. A. Allerman, E. A. Douglas, R. J. Kaplar, RF performance of $Al_{0.85}Ga_{0.15}N/Al_{0.70}Ga_{0.30}N$ high electron mobility transistors with 80-nm gates. *IEEE Electron Device Lett.* **40**, 17–20 (2019).

47. T. G. Ivanov, J. Weil, P. B. Shah, A. G. Birdwell, K. Kingkeo, E. A. Viveiros, "Diamond RF Transistor Technology with f_t= 41 GHz and f_{max}= 44 GHz," in *2018 IEEE/MTT-S International Microwave Symposium-IMS* (IEEE, 2018), pp. 1461–1463.

48. S. Imanishi, K. Horikawa, N. Oi, S. Okubo, T. Kageura, A. Hiraiwa, H. Kawarada, 3.8 W/mm RF power density for ALD Al_2O_3-based two-dimensional hole gas diamond MOSFET operating at saturation velocity. *IEEE Electron Device Lett.* **40**, 279–282 (2019).

49. C. J. Zhou, J. J. Wang, J. C. Guo, C. Yu, Z. Z. He, Q. B. Liu, X. D. Gao, S. J. Cai, Z. H. Feng, Radiofrequency performance of hydrogenated diamond MOSFETs with alumina. *Appl. Phys. Lett.* **114**, 063501 (2019).

50. X. Yu, W. Hu, J. Zhou, B. Liu, T. Tao, Y. Kong, T. Chen, Y. Zheng, 1 W/mm output power density for H-terminated diamond MOSFETs with Al_2O_3/SiO_2 bi-layer passivation at 2 GHz. *IEEE J. Electron Devices Soc.* **9**, 160–164 (2021).

51. K. Kudara, S. Imanishi, A. Hiraiwa, Y. Komatsuzaki, Y. Yamaguchi, Y. Kawamura, S. Shinjo, H. Kawarada, High output power density of 2DHG diamond MOSFETs with thick ALD-Al_2O_3. *IEEE Trans. Electron Devices* **68**, 3942–3949 (2021).

52. K. Kudara, M. Arai, Y. Suzuki, A. Morishita, J. Tsunoda, A. Hiraiwa, H. Kawarada, Over 1 A/mm drain current density and 3.6 W/mm output power density in 2DHG diamond MOSFETs with highly doped regrown source/drain. *Carbon* **188**, 220–228 (2022).

53. C. Yu, C. Zhou, J. Guo, Z. He, M. Ma, H. Yu, X. Song, A. Bu, Z. Feng, Hydrogen-terminated diamond MOSFETs on (0 0 1) single crystal diamond with state of the art high RF power density. *Funct. Diam.* **2**, 64–70 (2022).

54. Y. Wu, A. Saxler, M. Moore, R. P. Smith, S. Sheppard, P. M. Chavarkar, T. Wisleder, U. K. Mishra, P. Parikh, 30-W/mm GaN HEMTs by field plate optimization. *IEEE Electron Device Lett.* **25**, 117–119 (2004).

55. Y. Okamoto, Y. Ando, K. Hataya, T. Nakayama, H. Miyamoto, T. Inoue, M. Senda, K. Hirata, M. Kosaki, N. Shibata, M. Kuzuhara, Improved power performance for a recessed-gate AlGaN-GaN heterojunction FET with a field-modulating plate. *IEEE Trans. Microw. Theory Tech.* **52**, 2536–2540 (2004).

56. S. Kolluri, S. Keller, S. P. Denbaars, U. K. Mishra, N-polar GaN MIS-HEMTs with a 12.1-W/mm continuous-wave output power density at 4 GHz on sapphire substrate. *IEEE Electron Device Lett.* **32**, 635–637 (2011).

57. H. Lu, B. Hou, L. Yang, M. Zhang, L. Deng, M. Wu, Z. Si, S. Huang, X. Ma, Y. Hao, High RF performance GaN-on-Si HEMTs with passivation implanted termination. *IEEE Electron Device Lett.* **43**, 188–191 (2021).

58. S. Li, M. Wu, L. Yang, B. Yang, H. Sun, M. Zhang, B. Hou, H. Lu, X. Ma, Y. Hao, 15.1 W/mm power density GaN-on-GaN HEMT with high-gradient stepped-C doped buffer. *IEEE Electron Device Lett.* **46**, 365–368 (2025).

59. A. Bansal, R. Baby, A. Gowrisankar, V. S. Charan, R. Muralidharan, H. Chandrasekar, A. Sadhanala, S. Raghavan, D. N. Nath, Microwave power performance of buffer-free AlGaN/GaN MISHEMT with MOCVD grown ex situ SiN. *IEEE Trans. Electron Devices* **72**, 2226–2232 (2025).

60. Y. Zhang, F. Udrea, H. Wang, Multidimensional device architectures for efficient power electronics. *Nat. Electron.* **5**, 723–734 (2022).

61. A. Charnas, J. Anderson, J. Zhang, D. Zheng, D. Weinstein, P. D. Ye, Ultrathin indium oxide thin-film transistors with gigahertz operation frequency. *IEEE Trans. Electron Devices* **70**, 532–536 (2023).

62. D. Zheng, A. Charnas, J.-Y. Lin, J. Anderson, D. Weinstein, P. D. Ye, "Ultrathin Atomic-Layer-Deposited In_2O_3 Radio-Frequency Transistors with Record High f_T of 36 GHz and BEOL Compatibility," in *2023 IEEE Symposium on VLSI Technology and Circuits* (*VLSI Technology and Circuits*) (IEEE, 2023), pp. 1–2.

63. S. Li, M. Tian, Q. Gao, M. Wang, T. Li, Q. Hu, X. Li, Y. Wu, Nanometre-thin indium tin oxide for advanced high-performance electronics. *Nat. Mater.* **18**, 1091–1097 (2019).

64. Q. Hu, S. Zhu, C. Gu, S. Liu, M. Zeng, Y. Wu, Ultrashort 15-nm flexible radio frequency ITO transistors enduring mechanical and temperature stress. *Sci. Adv.* **8**, eade4075 (2022).

65. C. Tückmantel, U. Kalita, T. Haeger, M. Theisen, U. Pfeiffer, T. Riedl, Amorphous indium-gallium-zinc-oxide TFTs patterned by self-aligned photolithography overcoming the GHz threshold. *IEEE Electron Device Lett.* **41**, 1786–1789 (2020).

66. D. Zheng, A. Charnas, J. Anderson, H. Dou, Z. Hu, Z. Lin, Z. Zhang, J. Zhang, P.-Y. Liao, M. Si, H. Wang, D. Weinstein, P. D. Ye, "First demonstration of BEOL-compatible ultrathin atomiclayer-deposited InZnO transistors with GHz operation and record high bias-stress stability," in *2022 International Electron Devices Meeting* (*IEDM*) (IEEE, 2022), pp. 3–4.

67. J. Liu, Y. Zhou, J. Zhu, Y. Cai, K. M. Lau, K. J. Chen, DC and RF characteristics of AlGaN/GaN/InGaN/GaN double-heterojunction HEMTs. *IEEE Trans. Electron Devices* **54**, 2–10 (2007).

68. C.-W. Tsou, C.-Y. Lin, Y.-W. Lian, S. S. H. Hsu, 101-GHz InAlN/GaN HEMTs on silicon with high Johnson's figure-of-merit. *IEEE Trans. Electron Devices* **62**, 2675–2678 (2015).

69. S. Dai, Y. Zhou, Y. Zhong, K. Zhang, G. Zhu, H. Gao, Q. Sun, T. Chen, H. Yang, High f_T AlGa (In) N/GaN HEMTs grown on Si with a low gate leakage and a high ON/OFF current ratio. *IEEE Electron Device Lett.* **39**, 576–579 (2018).

70. W. Song, Z. Zheng, T. Chen, J. Wei, L. Yuan, K. J. Chen, RF linearity enhancement of GaN-on-Si HEMTs with a closely coupled double-channel structure. *IEEE Electron Device Lett.* **42**, 1116–1119 (2021).

71. Q. Yu, C. Shi, L. Yang, H. Lu, M. Zhang, M. Wu, B. Hou, F. Jia, F. Guo, X. Ma, Y. Hao, High current and linearity AlGaN/GaN/-graded-AlGaN: Si-doped/GaN heterostructure for low voltage power amplifier application. *IEEE Electron Device Lett.* **44**, 582–585 (2023).

72. T. Kamimura, Y. Nakata, M. Higashiwaki, Delay-time analysis in radio-frequency β-Ga_2O_3 field effect transistors. *Appl. Phys. Lett.* **117**, 253501 (2020).

73. A. Vaidya, C. N. Saha, U. Singisetti, Enhancement mode β-$(Al_xGa_{1-x})_2O_3/Ga_2O_3$ heterostructure FET (HFET) with high transconductance and cutoff frequency. *IEEE Electron Device Lett.* **42**, 1444–1447 (2021).

74. N. Kumar, D. Vaca, C. Joishi, Z. Xia, S. Rajan, S. Kumar, Ultrafast thermoreflectance imaging and electrothermal modeling of β-Ga_2O_3 MESFETs. *IEEE Electron Device Lett.* **41**, 641–644 (2020).

75. T. Favaloro, J.-H. Bahk, A. Shakouri, Characterization of the temperature dependence of the thermoreflectance coefficient for conductive thin films. *Rev. Sci. Instrum.* **86**, 024903 (2015).

76. K. Ghosh, U. Singisetti, Impact ionization in β-Ga_2O_3. *J. Appl. Phys.* **124**, 085707 (2018).

77. F. Zhou, H. Gong, M. Xiao, Y. Ma, Z. Wang, X. Yu, L. Li, L. Fu, H. H. Tan, Y. Yang, F.-F. Ren, S. Gu, Y. Zheng, H. Lu, R. Zhang, Y. Zhang, J. Ye, An avalanche-and-surge robust ultrawide-bandgap heterojunction for power electronics. *Nat. Commun.* **14**, 4459 (2023).

78. Silvaco, Atlas User's Manual Device Simulation Software, http://silvaco.com.

79. H. Fukui, Optimal noise figure of microwave GaAs MESFET's. *IEEE Trans. Electron Devices* **26**, 1032–1037 (1979).

80. D. Delagebeaudeuf, J. Chevrier, M. Laviron, P. Delescluse, A new relationship between the Fukui coefficient and optimal current value for low-noise operation of field-effect transistors. *IEEE Electron Device Lett.* **6**, 444–445 (1985).

81. S. Lardizabal, L. Dunleavy, W. Yau, S. Bar, “Experimental investigation of the temperature dependence of PHEMT noise parameters,” in *1994 IEEE MTT-S International Microwave Symposium Digest* (*Cat. No. 94CH3389-4*) (IEEE, 1994), pp. 845–848.

82. W. Lu, V. Kumar, R. Schwindt, E. Piner, I. Adesida, DC, RF, and microwave noise performances of AlGaN/GaN HEMTs on sapphire substrates. *IEEE Trans. Microw. Theory Tech.* **50**, 2499–2504 (2002).

83. H. K. Huang, C. S. Wang, Y. Wang, C. L. Wu, C. S. Chang, Temperature effects of low noise InGaP/InGaAs/GaAs PHEMTs. *Solid State Electron.* **47**, 1989–1994 (2003).

84. J. Lee, A. Kuliev, V. Kumar, R. Schwindt, I. Adesida, Microwave noise characteristics of AlGaN/GaN HEMTs on SiC substrates for broad-band low-noise amplifiers. *IEEE Microw. Wirel. Compon. Lett.* **14**, 259–261 (2004).

85. W. Lu, J. Yang, M. A. Khan, I. Adesida, AlGaN/GaN HEMTs on SiC with over 100 GHz f_T and low microwave noise. *IEEE Trans. Electron Devices* **48**, 581–585 (2001).

86. H. Sun, A. R. Alt, H. Benedickter, C. R. Bolognesi, High-performance 0.1-µm gate AlGaN/GaN HEMTs on silicon with low-noise figure at 20 GHz. *IEEE Electron Device Lett.* **30**, 107–109 (2009).

87. J. S. Moon, D. Wong, P. Hashimoto, M. Hu, I. Milosavljevic, P. Willadsen, C. McGuire, S. Burnham, M. Micovic, M. Wetzel, D. Chow, Sub-1-dB noise figure performance of high-power field-plated GaN HEMTs. *IEEE Electron Device Lett.* **32**, 297–299 (2010).

88. Z. H. Liu, G. I. Ng, S. Arulkumaran, Y. K. T. Maung, K. L. Teo, S. C. Foo, V. Sahmuganathan, T. Xu, C. H. Lee, High microwave-noise performance of AlGaN/GaN MISHEMTs on silicon with Al_2O_3 gate insulator grown by ALD. *IEEE Electron Device Lett.* **31**, 96–98 (2010).

89. Z. H. Liu, G. I. Ng, S. Arulkumaran, Y. Maung, K. L. Teo, S. C. Foo, S. Vicknesh, Temperature-dependent microwave noise characteristics in ALD Al_2O_3/AlGaN/GaN MISHEMTs on silicon substrate. *IEEE Electron Device Lett.* **32**, 318–320 (2011).

90. T. Huang, O. Axelsson, T. N. T. Do, M. Thorsell, D. Kuylenstierna, N. Rorsman, Influence on noise performance of GaN HEMTs with in situ and low-pressure-chemical-vapor-deposition SiN_x passivation. *IEEE Trans. Electron Devices* **63**, 3887–3892 (2016).

91. X. Liu, S. Zhang, K. Wei, J. Guo, X. He, Y. Zhang, H. Yin, S. Huang, X. Chen, Y. Zheng, X. Wang, S. Ouyang, Y. Li, 0.18 dB low-noise figure at 10 GHz for GaN MIS-HEMT with plasma-enhanced atomic layer deposition SiN layer. *IEEE Electron Device Lett.* **44**, 1080–1083 (2023).

92. A. Aleksov, A. Denisenko, U. Spitzberg, W. Ebert, E. Kohn, Microwave performance of diamond surface-channel FETs. *IEEE Electron Device Lett.* **23**, 488–490 (2002).

93. C. H. Lin, X. B. Mei, Y. C. Chou, L. S. Lee, J. M. Yang, M. Y. Nishimoto, P. H. Liu, R. To, A. Cavus, R. Tsai, M. Wojtowicz, R. Lai, "Sub-mW operation of InP HEMT X-band low-noise amplifiers for low power applications," in *2009 Annual IEEE Compound Semiconductor Integrated Circuit Symposium* (IEEE, 2009), pp. 1–4.

94. L. Liu, A. R. Alt, H. Benedickter, C. R. Bolognesi, InP-HEMT X-band low-noise amplifier with ultralow 0.6-mW power consumption. *IEEE Electron Device Lett.* **33**, 209–211 (2011).

95. D. C. Ruiz, T. Saranovac, D. Han, O. Ostinelli, C. R. Bolognesi, "Impact ionization control in 50 nm low-noise high-speed InP HEMTs with InAs channel insets," in *2019 IEEE International Electron Devices Meeting* (*IEDM*) (IEEE, 2019), pp. 3–9.
